# Proteomic aging clock predicts mortality and risk of common age-related diseases in diverse populations

**DOI:** 10.1038/s41591-024-03164-7

**Published:** 2024-08-08

**Authors:** M. Austin Argentieri, Sihao Xiao, Derrick Bennett, Laura Winchester, Alejo J. Nevado-Holgado, Upamanyu Ghose, Ashwag Albukhari, Pang Yao, Mohsen Mazidi, Jun Lv, Iona Millwood, Hannah Fry, Rodosthenis S. Rodosthenous, Jukka Partanen, Zhili Zheng, Mitja Kurki, Mark J. Daly, Aarno Palotie, Cassandra J. Adams, Liming Li, Robert Clarke, Najaf Amin, Zhengming Chen, Cornelia M. van Duijn

**Affiliations:** 1 Nuffield Department of Population Health, University of Oxford, Oxford, UK; 2 Analytic and Translational Genetics Unit, Massachusetts General Hospital, Boston, MA USA; 3 Program in Medical and Population Genetics, Broad Institute of MIT and Harvard, Boston, MA USA; 4 King Abdulaziz University and the University of Oxford Centre for Artificial Intelligence in Precision Medicine (KO-CAIPM), Jeddah, Saudi Arabia; 5 Department of Psychiatry, University of Oxford, Oxford, UK; 6 Biochemistry Department, Faculty of Science, King Abdulaziz University, Jeddah, Saudi Arabia; 7 Department of Epidemiology and Biostatistics, School of Public Health, Peking University, Beijing, China; 8 Peking University Center for Public Health and Epidemic Preparedness and Response, Beijing, China; 9 Key Laboratory of Epidemiology of Major Diseases (Peking University), Ministry of Education, Beijing, China; 10 Institute for Molecular Medicine Finland (FIMM), HiLIFE, University of Helsinki, Helsinki, Finland; 11 Research and Development, Finnish Red Cross Blood Service, Helsinki, Finland; 12 Centre for Medicines Discovery, Nuffield Department of Medicine, University of Oxford, Oxford, UK

**Keywords:** Predictive markers, Preventive medicine, Proteome informatics, Computational models, Machine learning

## Abstract

Circulating plasma proteins play key roles in human health and can potentially be used to measure biological age, allowing risk prediction for age-related diseases, multimorbidity and mortality. Here we developed a proteomic age clock in the UK Biobank (*n* = 45,441) using a proteomic platform comprising 2,897 plasma proteins and explored its utility to predict major disease morbidity and mortality in diverse populations. We identified 204 proteins that accurately predict chronological age (Pearson *r* = 0.94) and found that proteomic aging was associated with the incidence of 18 major chronic diseases (including diseases of the heart, liver, kidney and lung, diabetes, neurodegeneration and cancer), as well as with multimorbidity and all-cause mortality risk. Proteomic aging was also associated with age-related measures of biological, physical and cognitive function, including telomere length, frailty index and reaction time. Proteins contributing most substantially to the proteomic age clock are involved in numerous biological functions, including extracellular matrix interactions, immune response and inflammation, hormone regulation and reproduction, neuronal structure and function and development and differentiation. In a validation study involving biobanks in China (*n* = 3,977) and Finland (*n* = 1,990), the proteomic age clock showed similar age prediction accuracy (Pearson *r* = 0.92 and *r* = 0.94, respectively) compared to its performance in the UK Biobank. Our results demonstrate that proteomic aging involves proteins spanning multiple functional categories and can be used to predict age-related functional status, multimorbidity and mortality risk across geographically and genetically diverse populations.

## Abstract (web summary)

A proteomic aging clock, developed using data from the UK Biobank and validated using data from the China Kadoorie Biobank and FinnGen, predicts the incidence of 18 major chronic diseases, as well as multimorbidity and all-cause mortality.

## Main

Age is a major determinant for most common chronic diseases and causes of death^1,2^. Aging involves a progressive loss of physiological integrity and function over time, which ultimately leads to the development, and often co-occurrence, of major diseases and death. Rates of major chronic diseases such as ischemic heart disease (IHD), stroke, diabetes, liver and kidney diseases, neurodegenerative diseases and various cancers (for example, lung and colorectal) all increase with age^2–4^, although there is substantial variation across individuals in the timing and severity of age-related disorders. Chronological age is a strong but imperfect surrogate measure of ‘biological’ aging, which can be estimated more precisely by using ‘omics data to capture the level of biological functioning of an individual in comparison to an expected level of functioning for a given chronological age^5^.

How fast we age not only determines individual risk of major chronic diseases and premature death, but also shapes the extent of morbidity and disability in the population, which has a major impact on healthcare systems^2^. The ability to quantify, and possibly intervene upon, biological aging may therefore have important consequences for prevention of multimorbidity and premature death^6^. Some of the earliest and most successful biological aging clocks developed to date use DNA methylation (DNAm)^5,7^. Although DNAm can provide a window into epigenetic changes resulting from environmental exposures (for example, smoking^8^), protein levels may provide a more direct mechanistic and functional insight into aging biology^5^. Loss of proteostasis is a primary hallmark of aging^9^ and several previous studies have identified aging-related proteins (APs) and used these to develop proteomic age clocks^10–17^ to predict risk of certain diseases and mortality; however, none of these previous studies has developed a proteomic age clock in a large and well-powered general population sample that would allow a comprehensive assessment of the predictive performance of a proteomic clock across all major chronic diseases and age-related functional traits. Furthermore, to our knowledge none of these previous proteomic age clocks has been validated independently across diverse ancestry populations.

To address these gaps in knowledge, we used blood proteomic information to develop a proteomic age clock in a large sample of participants from the UK Biobank (UKB; *n* = 45,441) and validated this model in the China Kadoorie Biobank (CKB; *n* = 3,977) and FinnGen (*n* = 1,990), both of which are geographically and genetically distinct from the UK population. We then systematically assessed the influence of proteomic age gap (ProtAgeGap; defined as the difference between protein-predicted age and chronological age) on 27 aging-related phenotypes related to biological, functional and cognitive status; all-cause mortality; and incidence of 26 common age-related diseases that are either major causes of death or are highly prevalent in aging populations (rheumatoid arthritis, macular degeneration, osteoarthritis and osteoporosis).

## Results

### Proteomic age clock

**Fig. 1:**
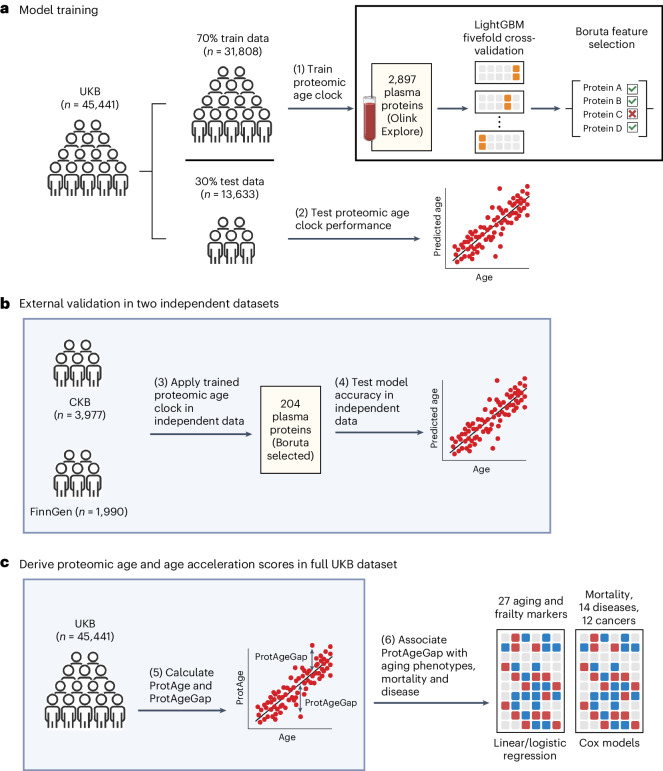
Overview of the study design and analytic approaches. **a**, UKB participants were split into training and test sets at a 70:30 ratio. In the training set, a LightGBM model was trained to predict chronological age using 2,897 plasma proteins and fivefold cross-validation. We identified 204 proteins relevant for predicting chronological age using the Boruta feature selection algorithm and retrained a refined LightGBM model using these 204 proteins, which was then evaluated in the UKB test set. **b**, Independent data from the CKB and FinnGen were used for further independent validation of the proteomic age clock model. **c**, Protein-predicted age (ProtAge) was calculated in the full UKB sample using fivefold cross-validation and LightGBM. ProtAgeGap was calculated as the difference between ProtAge and chronological age. We used linear and logistic regression to test associations between ProtAgeGap and a comprehensive panel of biological aging markers and measures of frailty and physical/cognitive status. Further, we used Cox proportional hazards models to test associations between ProtAgeGap and mortality, 14 common diseases and 12 cancers. Most association analyses were carried out only in the UKB, due to the smaller sample size in the CKB and the lack of disease cases in FinnGen. Figure created with BioRender.com.

A schematic representation of the study design and main analytic approaches is shown in Fig. 1. Characteristics of participants across the discovery (UKB) and two validation cohorts (CKB and FinnGen) are shown in Table 1. We used plasma proteomic expression data from the subset of 45,441 randomly selected UKB participants (54% female, age range 39–71 years), 3,977 CKB participants in an IHD case–cohort study (54% female, age range 30–78 years) and 1,990 Finnish (FinnGen) participants (52% female, age range 19–78 years). Across 11–16 years of follow-up in the UKB and 11–14 years of follow-up in the CKB, there were 4,828 (10.6%) and 1,426 (36%) deaths, respectively. Proteomic profiling was conducted among mostly healthy participants in FinnGen without major diseases and only 1% (*n* = 22) of FinnGen participants with proteomic data died during follow-up.

**Table 1:** Characteristics of study participants for each of the three cohorts

	UKB (*n* = 45,441)	CKB (*n* = 3,977)	FinnGen (*n* = 1,990)
**Age**			
Mean (s.d.)	57 (8.2)	57 (12)	56 (15)
Range (years)	39–71	30–78	19–78
**Sex**			
Female	24,579 (54.1%)	2,137 (53.7%)	1,032 (51.9%)
**BMI (kg** **m**^**−**^^**2**^**)**			
Mean (s.d.)	27 (4.8)	24 (3.6)	26 (4.5)
**Ethnicity**			
White	42,320 (93.1%)	–	–
Asian	1,016 (2.2%)	–	–
Black	1,114 (2.5%)	–	–
Mixed	293 (0.6%)	–	–
Other	554 (1.2%)	–	–
**Geographic region**			
Gansu (rural)	–	397 (10.0%)	–
Haikou (urban)	–	298 (7.5%)	–
Harbin (urban)	–	598 (15.0%)	–
Henan (rural)	–	493 (12.4%)	–
Hunan (rural)	–	462 (11.6%)	–
Liuzhou (urban)	–	379 (9.5%)	–
Qingdao (urban)	–	415 (10.4%)	–
Sichuan (rural)	–	341 (8.6%)	–
Suzhou (urban)	–	252 (6.3%)	–
Zhejiang (rural)	–	342 (8.6%)	–
**Incident diabetes**			
Yes	2,781 (6.1%)	160 (4.0%)	–
**Incident IHD**			
Yes	4,546 (10.0%)	2,121 (53.3%)	–
**Incident all stroke**			
Yes	1,362 (3.0%)	566 (14.2%)	–
**Incident ischemic stroke**			
Yes	1,182 (2.6%)	521 (13.1%)	–
**Incident COPD**			
Yes	2,059 (4.5%)	178 (4.5%)	–
**Incident chronic liver diseases**			
Yes	1,011 (2.2%)	39 (1.0%)	–
**Incident CKDs**			
Yes	2,626 (5.8%)	52 (1.3%)	–
**All-cause mortality**			
Dead	4,828 (10.6%)	1,426 (35.9%)	22 (1.1%)

Hyphens are shown for data categories that are not applicable in a specific cohort or for which there were not enough cases.

**Fig. 2:**
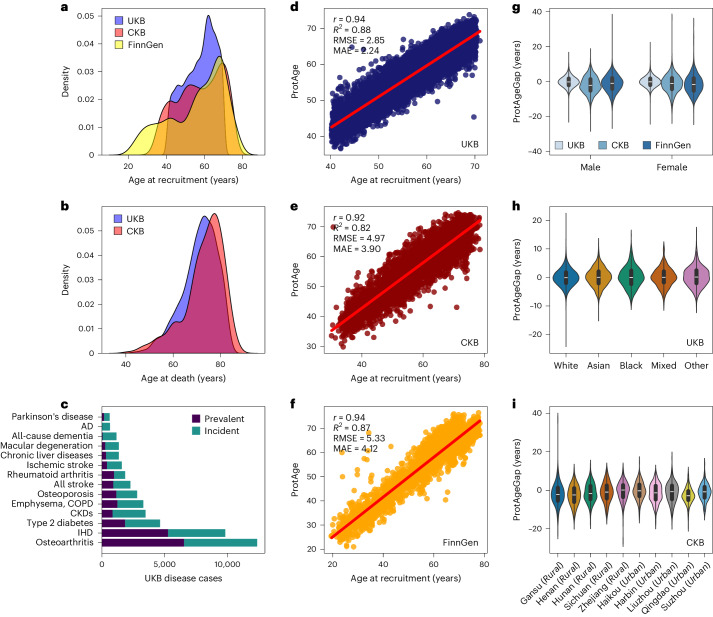
Proteomic aging clock performance across cohorts. **a**, Density plot of age at recruitment in the UKB, CKB and FinnGen. **b**, Density plot of age at death in the UKB (4,784 deaths; 10.6%) and CKB (1,426 deaths; 36%). **c**, Counts of prevalent and incident cases of all common diseases studied in the UKB sample (*n* = 45,441). **d**, Performance of the trained proteomic aging model in the UKB holdout test set (*n* = 13,633). **e**, Performance of the trained proteomic aging model in the CKB (*n* = 3,977). **f**, Performance of the trained proteomic aging model in FinnGen (*n* = 1,990). **g**, Sex distributions of ProtAgeGap in the UKB (female *n* = 24,579; male *n* = 20,862), CKB (female *n* = 2,137; male *n* = 1,840) and FinnGen (female *n* = 1,032; male *n* = 958). **h**, Distributions of ProtAgeGap according to self-reported ethnicity in the UKB (white *n* = 42,320; Black *n* = 1,114; Asian *n* = 1,016; other *n* = 554; mixed *n* = 293). **i**, Distributions of ProtAgeGap according to geographic region of residence in the CKB (Gansu *n* = 397; Henan *n* = 493; Hunan *n* = 462; Sichuan *n* = 341; Zhejiang *n* = 342; Haikou *n* = 298; Harbin *n* = 598; Liuzhou *n* = 379; Qingdao *n* = 415; Suzhou *n* = 252). Correlation coefficients shown in **d**–**f** are Pearson correlation coefficients. Violin plots in **g**–**i**, with center line, box limits and whiskers representing the median, interquartile range and minima/maxima within each group, respectively. RMSE, root mean squared error; MAE, mean absolute error.

We randomly split the UKB cohort into 70% training and 30% test sets to develop the proteomic age clock. In the training phase (*n* = 31,808), we compared six machine-learning methods (LASSO, elastic net, gradient boosting and three neural networks) to train proteomic age clock models to predict chronological age using normalized expression of 2,897 proteins from the Olink Explore 3072 panel. We found that gradient boosting (LightGBM^18^) showed the second-best age prediction accuracy in the UKB test set (*n* = 13,633) but the highest accuracy in the independent samples from the CKB and FinnGen (Supplementary Fig. 1). Based on its superior generalizability, we selected LightGBM as our final model and used the Boruta^19^ feature selection algorithm and SHAP (SHapley Additive exPlanations)^20^ values to identify the subset of all proteins relevant for predicting chronological age (Methods). This process resulted in the identification of 204 APs in our dataset (Supplementary Table 1). The correlation structure among these proteins is shown in Supplementary Fig. 2. Protein-predicted age (ProtAge) from this 204-protein model explained a similar degree of variation in chronological age compared to the 2,897-protein model (Supplementary Fig. 3a,b), with similar model error across different age groups (Supplementary Fig. 4). Our gradient-boosting ProtAge model explained a high degree of variation in chronological age in the UKB test set (*R*^2^ = 0.88; Pearson *r* = 0.94) and in the independent validation sets from the CKB (*R*^2^ = 0.82; Pearson *r* = 0.92) and FinnGen (*R*^2^ = 0.87; Pearson *r* = 0.94) (Fig. 2d–f).

**Extended Data Fig. 1:**
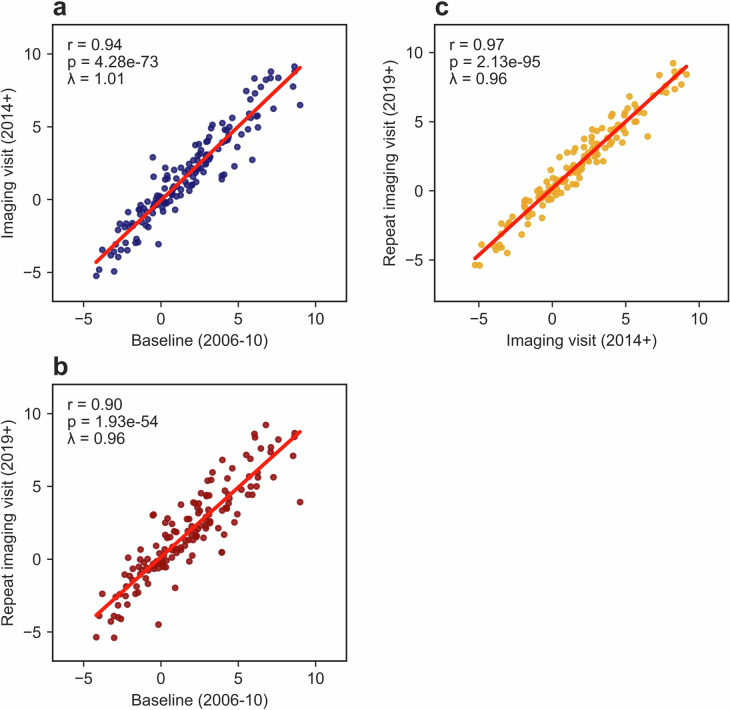
Stability of ProtAge protein associations with age across three time points. Comparison of betas for the association between age and each of the 149 ProtAge APs with repeat measurements available during baseline and two follow-up imaging visits (n = 1,085). **a)** Comparison of betas for the association between age and each of the 149 ProtAge APs during baseline and the 2014+ follow-up imaging visit. **b)** Comparison of betas for the association between each of these 149 ProtAge APs and age during baseline and the 2019+ imaging visit. **c)** Comparison of betas for the association between each of the 149 ProtAge APs and age during the 2014+ imaging visit and during the 2019+ imaging visit. Shown in each plot are the Pearson correlation coefficient (r), p-value for the correlation, and the model slope (λ). APs: aging-related proteins.

To assess whether each of our AP’s association with age was stable over time, we used repeat protein expression measurements available for a subset of 149 ProtAge proteins among 1,085 UKB participants who had proteomic data measured at three time points (baseline (2006 to 2011), imaging study visit (2014+) and repeat imaging visit (2019+)). For each of these 149 APs, we assessed their association with age at each study visit using linear regression. Beta coefficients for the associations of these APs with age across all three time points were strongly correlated with each other (Pearson *r* = 0.90–0.97), suggesting good stability of associations between APs and age across repeat visits spanning at least 9–13 years (Extended Data Fig. 1).

Using 204 APs in the final model, we calculated participants’ ProtAgeGap as the difference between ProtAge and chronological age in all three cohorts. In the UKB, the average years of ProtAgeGap among the top 5% and bottom 5% of ProtAgeGap was 6.3 and −6 years, respectively, resulting in a mean difference of approximately 12.3 years in biological aging between them. ProtAgeGap showed similar distributions across all three cohorts in females and males, across self-reported ethnicities in the UKB and across geographical regions in the CKB (Fig. 2g–i).

As a final feature selection step, we employed recursive feature elimination using SHAP values (Methods) to identify a model of 20 proteins (ProtAge20) that achieved 95% of the age prediction performance of the 204-protein model (Pearson *r* = 0.89, *R*^2^ = 0.78; Supplementary Fig. 3c,d and Supplementary Table 2). Correlation among these 20 proteins is shown in Supplementary Fig. 5. We further calculated the proteomic age gap according to these top 20 proteins (ProtAgeGap20) in the UKB using the same approach as above. In models with fewer than 20 proteins, model performance dropped precipitously (Supplementary Fig. 3d).

### Proteomic aging predicts frailty and aging phenotypes

**Fig. 3:**
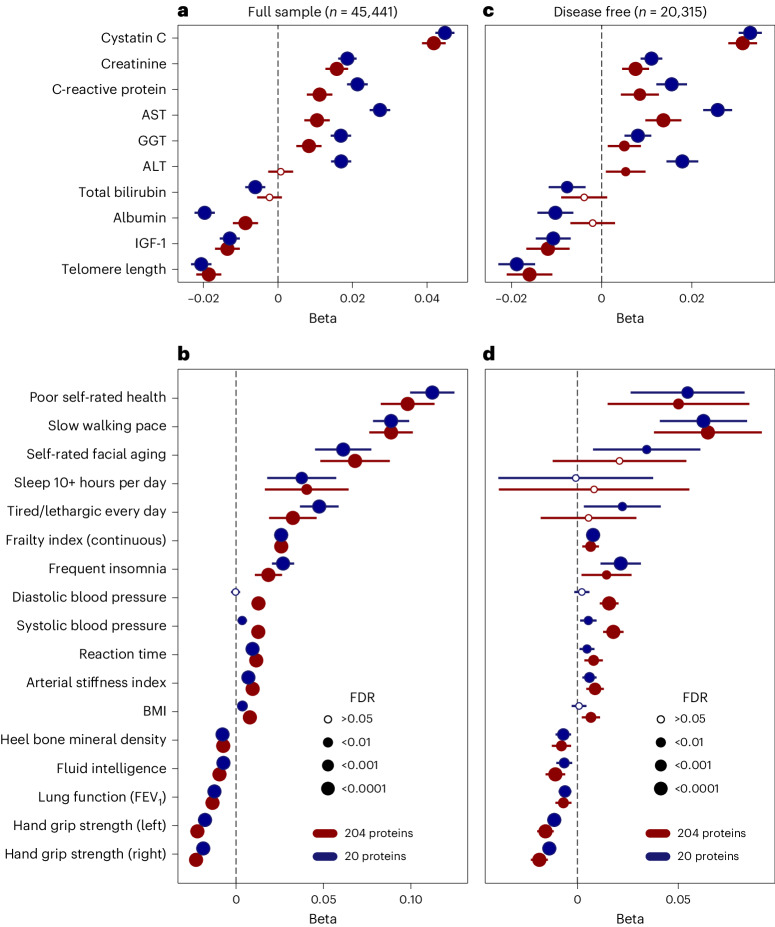
ProtAgeGap is associated with age-related biological, physical and cognitive function. **a**, Associations between ProtAgeGap and biological aging mechanisms in the full UKB sample (*n* = 45,441). **b**, Associations between ProtAgeGap and measures of physiological and cognitive (reaction time and fluid intelligence) function in the full UKB sample (*n* = 45,441). **c**, Associations between ProtAgeGap and biological aging mechanisms in the subsample of UKB participants with no lifetime diagnosis of any of the 26 diseases studied (*n* = 20,315). **d**, Associations between ProtAgeGap and measures of physiological and cognitive function in the subsample of UKB participants with no lifetime diagnosis of any of the 26 diseases studied (*n* = 20,315). All models used linear or logistic regression and were adjusted for age, sex, Townsend deprivation index, recruitment center, ethnicity, IPAQ activity group and smoking status. In all plots, beta estimates (and 95% confidence intervals) for the association between ProtAgeGap and each outcome are shown on the *x* axis. Beta estimates in red are from the full 204-protein model (ProtAgeGap), whereas beta estimates in blue are from the smaller proteomic age clock model with 20 proteins (ProtAgeGap20). FEV_1_, forced expiratory volume in 1 s; IPAQ, International Physical Activity Questionnaire; FDR, false discovery rate.

To understand how proteomic aging may influence aging-related physiological and cognitive function, we examined associations in the UKB of ProtAgeGap with (1) a comprehensive frailty index^21^ (Methods); (2) 16 individual measures of physical (for example, slow walking pace and grip strength) and cognitive function (reaction time and fluid intelligence); and (3) ten measures of biological aging (for example, telomere length and insulin-like growth factor 1 (IGF-1)) and clinical blood biochemistry (for example, albumin and creatinine). After adjustment for chronological age, sex and major sociodemographic and lifestyle confounders, ProtAgeGap was significantly associated with all measures investigated except for two liver biomarkers (alanine aminotransferase (ALT) and total bilirubin; Fig. 3a,b). Among the biological aging mechanisms investigated (Fig. 3a), increasing ProtAgeGap was associated with increasing levels of two kidney function biomarkers (cystatin C and creatinine), two liver enzymes (aspartate aminotransferase (AST), γ-glutamyl transferase (GGT)) and C-reactive protein; and was associated with decreased levels of albumin, IGF-1 and telomere length. Among physical measures (Fig. 3b), increasing ProtAgeGap was associated with poor self-rated health, slow walking pace, self-rating one’s face as older than average, sleeping ≥10 h per day, feeling tired every day and having frequent insomnia. Increasing ProtAgeGap was also associated with higher values of a frailty index, systolic and diastolic blood pressure, reaction time, arterial stiffness and body mass index (BMI); and with lower values of bone mineral density, fluid intelligence, lung function and hand grip strength.

To explore whether these associations are explained by reverse causation (resulting from a nondetected pathology), we also restricted the analyses to a subset of UKB participants who had no lifetime diagnoses (according to hospital inpatient, cancer registry and primary care records) of any of the 26 diseases studied (*n* = 20,315). Among these participants (Fig. 3c,d), we found that ProtAgeGap remained significantly associated with nearly all markers except for albumin (which is a typical protein marker of end-stage morbidity), total bilirubin, self-rated facial aging, sleeping for 10+ hours per day and feeling tired every day (Fig. 3d).

ProtAgeGap20 was associated with all aging functional phenotypes except for diastolic blood pressure. Compared to the 204-protein model, ProtAgeGap20 showed stronger effect estimates in relation to biological measures of aging (for example, telomeres and albumin) (Fig. 3a) but somewhat smaller effect estimates for measures of frailty and physiological/cognitive function (Fig. 3b). ProtAgeGap20 was significantly associated with all biological aging markers (Fig. 3c) in the subset of UKB participants without lifetime disease diagnoses and was associated with all physiological measures except sleeping for 10+ hours per day, diastolic blood pressure and BMI (Fig. 3d). Summary statistics from all models are shown in Supplementary Tables 3–6.

### Proteomic aging is a strong predictor of common diseases

**Fig. 4:**
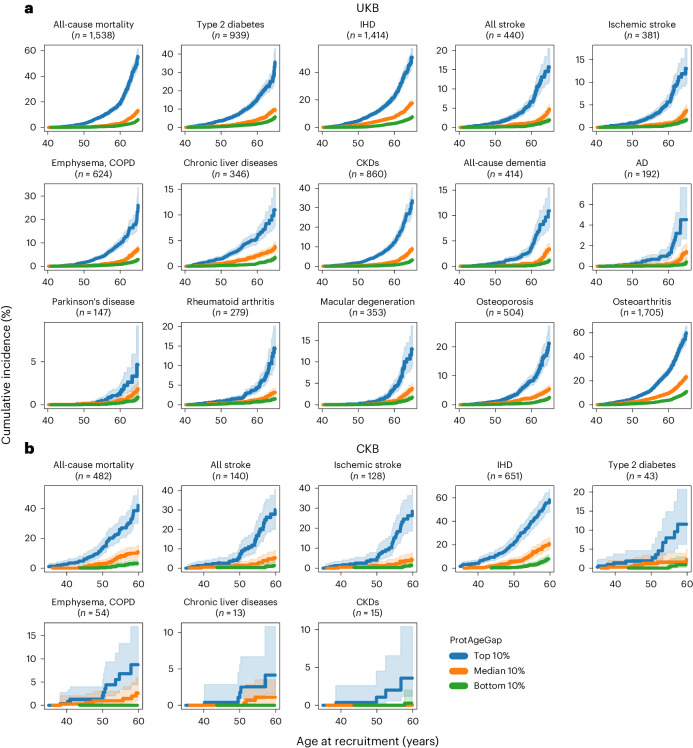
ProtAgeGap stratifies individuals into divergent age-specific mortality and disease risk trajectories in the UKB and CKB. **a**,**b**, Cumulative incidence plots for the indicated diseases and mortality for the top, median and bottom deciles of ProtAgeGap in the UKB (*n* = 45,441) (**a**) and CKB (*n* = 3,977) (**b**). The number of incident cases is shown for each disease, indicating the total number of incident cases only among individuals in the three deciles shown, not the full dataset. Incidence rates are shown for the subsequent 11–16 years (UKB) or 11–14 years (CKB) of follow-up after recruitment for each given age at recruitment (for example, in **a** the cumulative incidence rate shown at age 65 years is the rate of incident cases in the 11–16 years of follow-up for those aged 65 years at recruitment). All plots show the cumulative density of events at a given timepoint based on the Kaplan–Meier survival function, with 95% confidence intervals shown in lighter shading. Diseases shown here for the CKB are those with more than 10 cases across the three deciles of ProtAgeGap.

UKB participants in the top, median and bottom deciles of ProtAgeGap showed divergent age-specific incidence rates of all-cause mortality and the 14 common noncancer diseases studied (Fig. 4a and Supplementary Table 16). Cumulative incidence trajectories according to these deciles of ProtAgeGap were similar in females and males (Supplementary Figs. 6 and 7). For those aged 65 years at recruitment, the highest cumulative incidence rates (equivalent to absolute risk) across the study follow-up period of 11–16 years for the top decile of ProtAgeGap were observed for osteoarthritis (59.4%), all-cause mortality (55.2%), IHD (50.6%), type 2 diabetes (35.3%) and chronic kidney disease (CKD; 33.6%). Neurodegenerative diseases (Parkinson’s disease, all-cause dementia and Alzheimer’s disease (AD)) all showed cumulative incidence rates below 1% in the bottom decile of ProtAgeGap across all recruitment ages, which may in part be explained by the fact that onset is typically at older ages for these diseases.

In the CKB, we also calculated cumulative incidence rates according to deciles of ProtAgeGap for diseases with >10 incident cases across the three deciles of ProtAgeGap (Fig. 4b and Supplementary Table 17). We observed clear differences for IHD, all-cause mortality, all stroke and ischemic stroke. Differences were also observed for type 2 diabetes, chronic obstructive pulmonary disease (COPD), chronic liver diseases and CKD; however, confidence intervals were much wider due to a smaller number of incident cases.

**Fig. 5:**
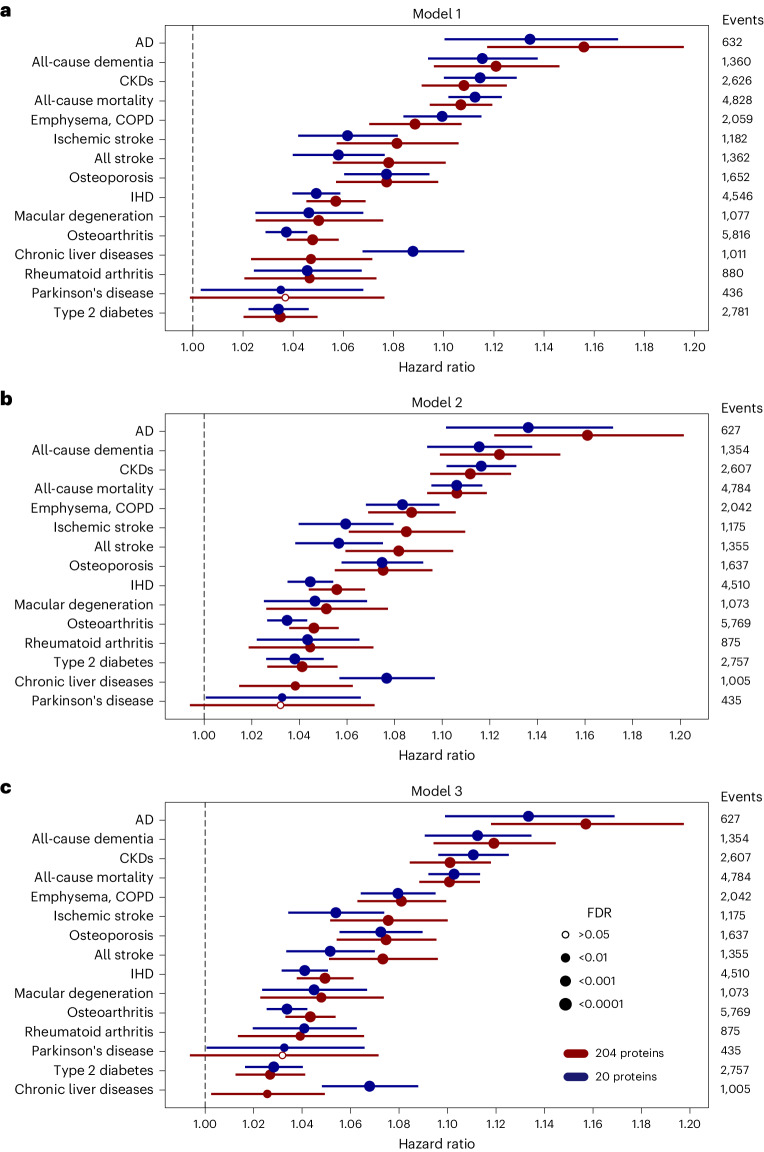
Effect sizes of the associations of ProtAgeGap with mortality and common diseases are largely invariant to covariate adjustment. Associations between ProtAgeGap and mortality and disease incidence using Cox proportional hazards models are shown for models with increasing levels of covariate adjustment. Shown on the *x* axis are HRs (and 95% CIs) for the effect of ProtAgeGap on the outcomes shown. Events listed are the total number of incident cases for each outcome. All models were run using the full UKB sample (*n* = 45,441). **a**, Model 1 was adjusted for age and sex. **b**, Model 2 was adjusted for age, sex, ethnicity, Townsend deprivation index, recruitment center, IPAQ activity group and smoking status. **c**, Model 3 was adjusted for age, sex, ethnicity, Townsend deprivation index, recruitment center, IPAQ activity group, smoking status, BMI and prevalent hypertension. HR estimates in red are from the full 204-protein model (ProtAgeGap), whereas estimates in blue are from the smaller proteomic age clock model with 20 proteins (ProtAgeGap20).

We further used multivariable Cox proportional hazards models to investigate whether associations of ProtAgeGap with mortality and the 14 common noncancer diseases studied persisted after adjustment for chronological age, sex, smoking, physical activity, sociodemographic factors and clinical risk factors. ProtAgeGap showed a significant association with mortality and all noncancer incident disease outcomes except Parkinson’s disease across all models in the UKB (Fig. 5). In the fully adjusted model that also included covariates for BMI and prevalent hypertension (model 3), the largest effect size per 1-year increase of ProtAgeGap was observed for AD (hazard ratio (HR) 1.16; 95% confidence interval (CI) 1.12–1.20), all-cause dementia (HR 1.12; 95% CI 1.10–1.15) and CKD (HR 1.10; 95% CI 1.08–1.11). ProtAgeGap20 was associated with all diseases investigated, including Parkinson’s disease. Summary statistics from all models are shown in Supplementary Tables 7–12.

Based on these HRs (reported per 1-year increase of ProtAgeGap), we estimated that those in the top 5% of ProtAgeGap had on average a 2.6-fold higher risk of AD compared to those with no difference between ProtAge and chronological age and a 5.8-fold higher risk of AD compared to those in the bottom 5% of ProtAgeGap. For CKD, the increases in risk were 1.8-fold (top 5% versus 0) and 3.1-fold (top 5% versus bottom 5%) and for mortality the increases in risk were 1.9-fold (top 5% versus 0) and 3.6-fold (top 5% versus bottom 5%).

**Extended Data Fig. 2:**
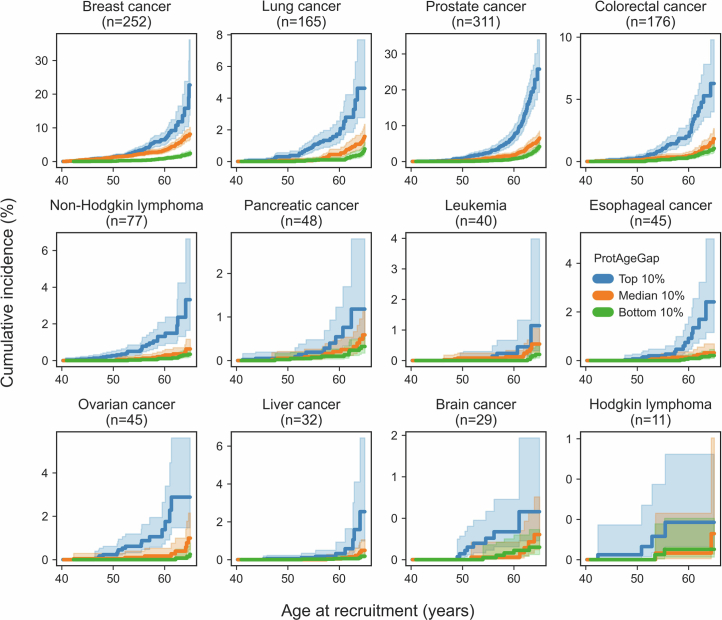
ProtAgeGap and age-specific cancer risk trajectories in the UKB. Cumulative incidence plots for the top, median, and bottom deciles of ProtAgeGap in the UK Biobank (UKB; n = 45,441). Number of incident cases are shown for each cancer – these numbers reflect the total number of incident cases present only among those in the 3 deciles shown, not the full dataset. Incidence rates are for the 11-16 years after recruitment. Incidence rates are shown for the subsequent 11-16 years of follow up after recruitment for each given age at recruitment (for example, the cumulative incidence rate shown at age 65 is the rate of incident cases in the 11-16 years of follow up for those aged 65 at recruitment). All plots show the cumulative density of events at a given timepoint based on the Kaplan-Meier survival function, with 95% confidence intervals in lighter shading. Certain plots show multiple 0s on the y-axis because these represent decimal values < 0.5 and the y-axis values are rounded to a single digit. ProtAgeGap: proteomic age gap (in years).

**Extended Data Fig. 3:**
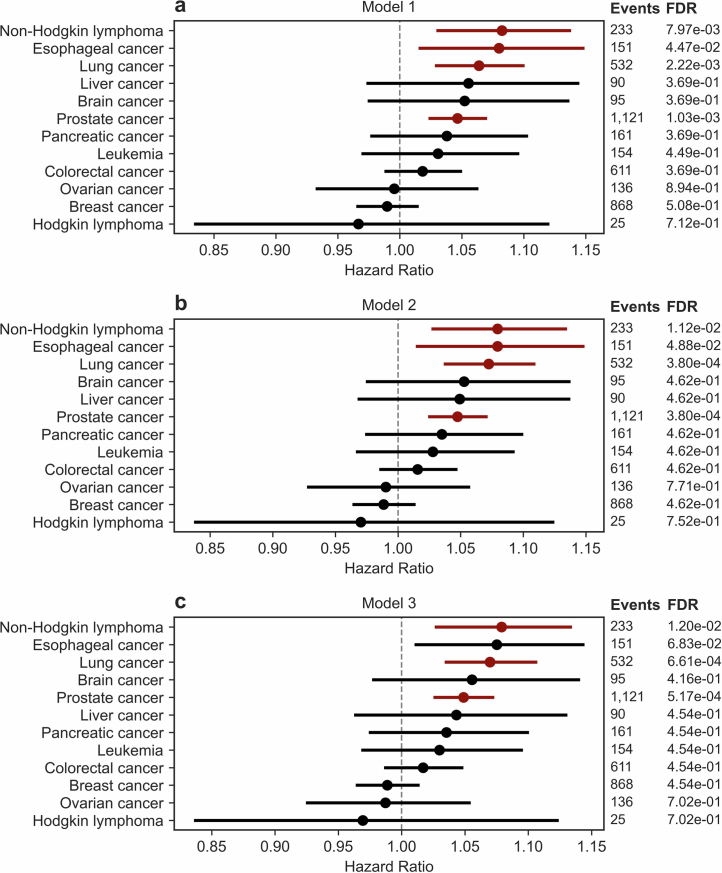
Associations between ProtAgeGap and cancers in the UKB. Associations between ProtAgeGap and and incident cancer diagnoses in Cox proportional hazards models with increasing levels of covariate adjustment. Shown on the x-axis are hazard ratios (and 95% confidence intervals) for the effect of ProtAgeGap on the outcomes shown. Events listed are the total number of incident cases for each outcome. Within each model, p-values across tests for all outcomes were corrected for multiple comparisons using the false discovery rate (FDR). All models were run in the UK Biobank (UKB; n = 45,441). **a)**. Model 1 is adjusted for age and sex. **b)** Model 2 is adjusted for age, sex, Townsend deprivation index, recruitment centre, IPAQ activity group, and smoking status. **c)** Model 3 is adjusted for age, sex, Townsend deprivation index, recruitment centre, IPAQ activity group, smoking status, BMI, and prevalent hypertension. ProtAgeGap: proteomic age gap (in years).

In the UKB, we also investigated the cumulative incidence of cancer diagnoses according to deciles of ProtAgeGap (Extended Data Fig. 2), with clear differences observed for eight cancers (breast, lung, prostate, colorectal, non-Hodgkin lymphoma, esophageal, ovarian and liver). In Cox multivariable models, ProtAgeGap was associated with four cancers (esophageal, lung, non-Hodgkin lymphoma and prostate) after adjustment for age, sex, sociodemographic and lifestyle factors, BMI and prevalent hypertension (Extended Data Fig. 3). Summary statistics are shown in Supplementary Tables 13–15.

**Extended Data Fig. 4:**
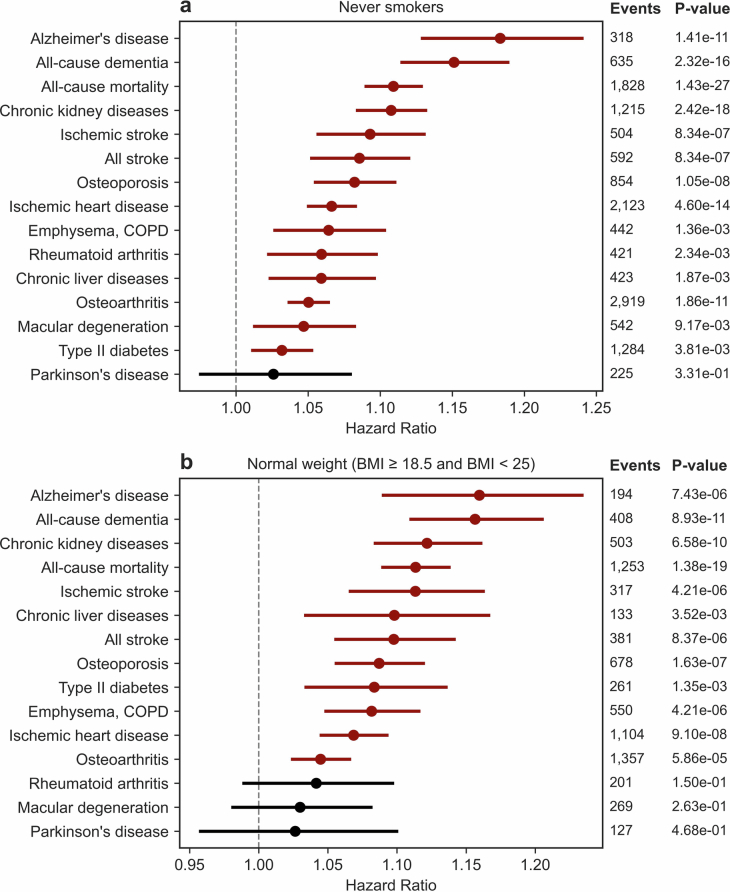
Effect size of ProtAgeGap on mortality and disease among non-smokers and those within normal weight range. Associations between ProtAgeGap and mortality or diseases among UK Biobank participants who report being never smokers (n = 24,528) **(a)** and with a BMI ≥ 18.5 and < 25 kg/m^2^ (n = 14,555) **(b)**. Shown on the x-axis are hazard ratios (and 95% confidence intervals) for the effect of ProtAgeGap on the outcomes shown. Events listed are the total number of incident cases for each outcome. All models are Cox proportional hazards models using model 2 (adjusted for age, sex, Townsend deprivation index, recruitment centre, and IPAQ activity group). No adjustment was made for multiple comparisons. ProtAgeGap: proteomic age gap (in years).

Although the analyses described above were adjusted for smoking status, we conducted further sensitivity analyses in never smokers. Among never smokers, ProtAgeGap remained significantly associated with mortality and all noncancer outcomes, except Parkinson’s disease (Extended Data Fig. 4a). In a similar sensitivity analysis restricted to those within a normal weight range (BMI ≥ 18.5 and BMI < 25), ProtAgeGap remained significantly associated with all outcomes except Parkinson’s disease, macular degeneration and rheumatoid arthritis (Extended Data Fig. 4b).

**Extended Data Fig. 5:**
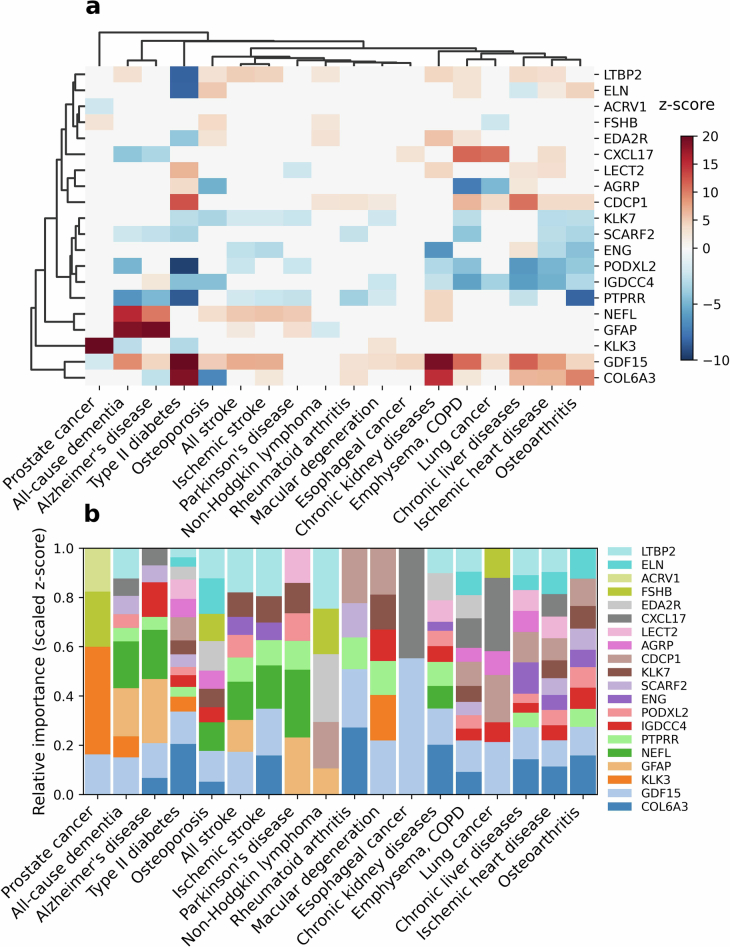
Associations between individual ProtAge APs and each disease studied. For each outcome associated with ProtAgeGap20, a Cox proportional hazards model (n = 45,441) was calculated with all 20 proteins from the ProtAgeGap20 score, adjusted for age, sex (except prostate cancer), ethnicity, Townsend deprivation index, recruitment center, IPAQ activity group, and smoking status. No adjustment was made for multiple comparisons. In **a)**, the association between each protein and incident disease is colored by z-score, with z-scores for associations with p-value ≥ 0.05 set to 0. In **b)**, the importance of each protein with p < 0.05 is shown as a relative contribution. Relative contribution for each disease is calculated by scaling z-score for significant proteins such that they add to 1. APs: aging-related proteins; ProtAgeGap20: proteomic age gap from the 20-protein model.

For the 20 proteins included in ProtAge20, we used Cox models to further assess associations of the individual proteins with all ProtAgeGap20-associated diseases. Notably, GDF15 was associated with 16 of 18 diseases studied, whereas ACRV1 (a testis-specific protein involved in spermatogenesis) was only associated with prostate cancer (Extended Data Fig. 5a). In Extended Data Fig. 5b, the relative weight of each protein’s association with each disease is shown as a scaled *z*-score. As expected from an aging signature, many of the proteins that make up the proteomic age clock are associated with multiple major chronic diseases.

### Proteomic aging increases with increasing multimorbidity

**Extended Data Fig. 6:**
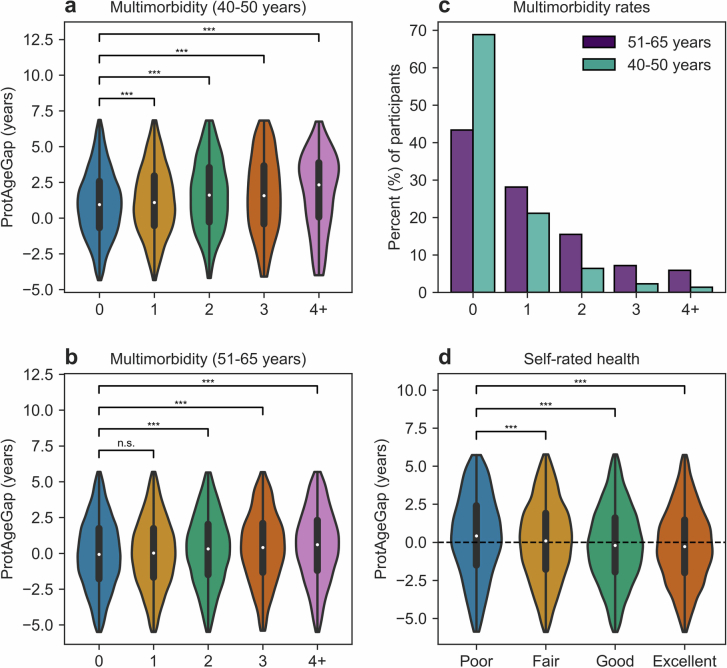
ProtAgeGap increases linearly with increasing disease multimorbidity. **a)** Years of ProtAgeGap in those with 0 (n = 6,826), 1 (n = 2,056), 2 (n = 605), 3 (n = 206), and 4+ (n = 116) comorbid conditions among UK Biobank (UKB) participants 40-50 years old at recruitment (total n = 9,809). **b)** Years of ProtAgeGap in those with 0 (n = 10,665), 1 (n = 6,903), 2 (n = 3,765), 3 (n = 1,702), and 4+ (n = 1,410) comorbid conditions among UKB participants aged 51-65 years old at recruitment (total n = 24,445). **c)** Percentages of the UKB population with 0, 1, 2, 3, and 4+ lifetime disease diagnoses. **d)** Years of ProtAgeGap according to levels of self-rated health in the UKB (total n = 43,393; Poor n = 2,249; Fair n = 9,355; Good n = 24,752; Excellent n = 7,004). Multimorbidity is defined as the number of lifetime diagnoses of any of the 26 diseases analyzed in this study. In **a**, **b**, and **d**, violin plots with center line, box limits, and whiskers represent the median, interquartile range, and minima/maxima within each group. For violin plots only, outliers were trimmed that were more than 2 standard deviations from total mean across all groups in the population subgroup plotted. Tests for significant differences between the means of groups were performed using a two-sided t-test. n.s.: not statistically significant; *** p-value < 0.001; ProtAgeGap: proteomic age gap (in years).

We defined multimorbidity as the number of lifetime diagnoses of any of the 26 diseases examined in the UKB and categorized participants according to having 0, 1, 2, 3 or 4+ lifetime diagnoses. We found that the average years of ProtAgeGap increased with number of lifetime conditions (Extended Data Fig. 6). We also found that this effect was more pronounced for younger participants at recruitment (aged 40–50 years; Extended Data Fig. 6a), among whom the presence of disease was less common (Extended Data Fig. 6c). On average, 1.5 more years of ProtAgeGap was observed in those with 4+ lifetime diagnoses compared to those with 0 diagnoses in participants aged 40–50 years at recruitment (Extended Data Fig. 6a), whereas in those aged 51–65 years at recruitment we observed 0.8 more years of ProtAgeGap (Extended Data Fig. 6b). The relationship between ProtAgeGap and multimorbidity status derived from health records was also reflected in self-reported health information. On average, 0.9 fewer years of ProtAgeGap was observed in those reporting excellent health (likely no diseases present) compared to those with poor self-reported health (Extended Data Fig. 6d).

### Biological functions and protein interaction networks

**Extended Data Fig. 7:**
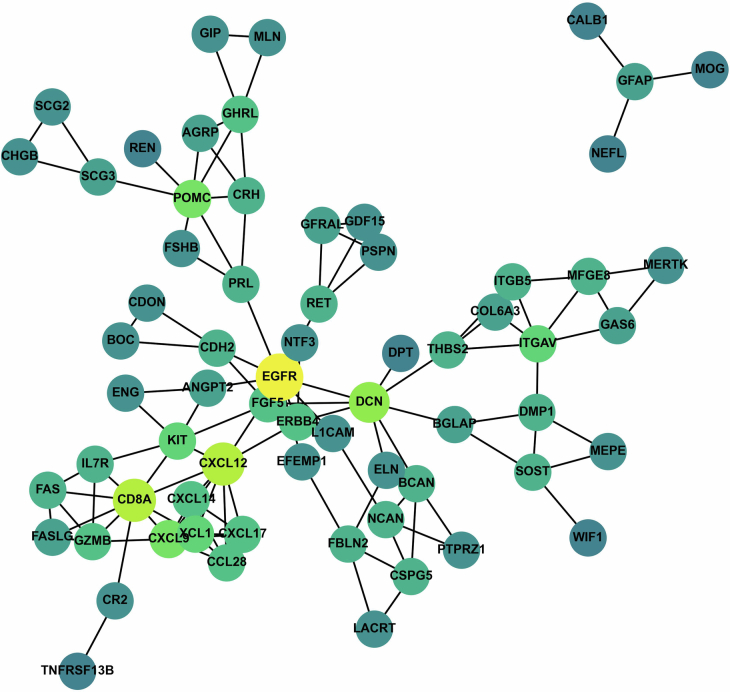
Protein–protein interaction network of ProtAge APs from the STRING database. Protein–protein interaction (PPI) network of a highly interconnected subset of APs in the ProtAge model with at least 2 node connections using experimental PPI information from the STRING database. Proteins are sized and colored by number of connections, with those showing a greater number of connections with other proteins displayed larger and more yellow.

Testing for functional enrichment among the 204 APs revealed that these APs were enriched for one Gene Ontology (GO) biological process: anatomical structure development and developmental process. No enrichments were found using GO molecular function, Kyoto Encyclopedia of Genes and Genomes (KEGG) or Reactome; however, these 204 APs showed a highly interconnected subnetwork of 66 proteins with at least two node connections in a protein–protein interaction (PPI) network using coexpression information from the STRING database (Extended Data Fig. 7). Individual proteins with the greatest numbers of connections to other proteins were EGFR (involved in cancer drug resistance, brain structure and platelet count), CXCL12 (an immune-related chemokine involved in immune surveillance, inflammation response, tissue homeostasis and tumor growth and metastasis), ITGAV (an integrin protein implicated in body height, handedness, dyslexia and albumin/creatinine metabolism), CXCL9 (implicated in T cell function and inflammation) and CD8A (a CD8 antigen implicated in the innate immune system).

**Extended Data Fig. 8:**
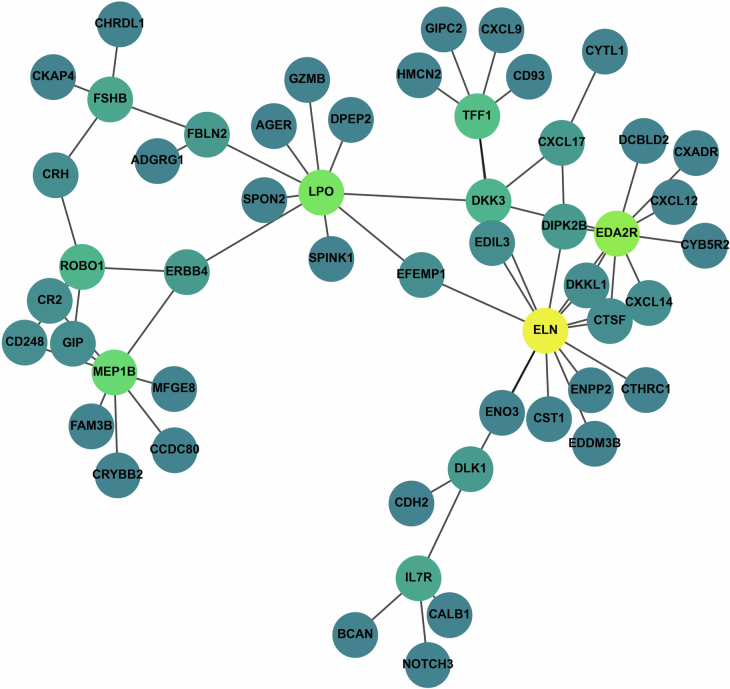
Protein–protein interaction network of ProtAge APs using SHAP interaction values. Protein–protein interaction (PPI) network using SHAP values from the trained ProtAge model. Proteins shown are only those that are highly interconnected using a cutoff of 0.0083 for mean absolute SHAP interaction values. Proteins are sized and colored by number of connections, with those showing a greater number of connections with other proteins displayed larger and more yellow.

We also used SHAP interaction values from our trained ProtAge model to calculate a second PPI network that represents the interactions of proteins together in the model to predict age (Extended Data Fig. 8). Individual proteins with the largest numbers of connections to other proteins according to SHAP interaction values were ELN (an elastic fiber protein that makes up part of the extracellular matrix and confers elasticity to organs and tissues, including the heart, skin, lungs, ligaments and blood vessels), EDA2R (involved in the NF-κB and innate immune pathways and implicated in baldness, estradiol, testosterone and HDL metabolism), LTPB2 (a protein involved in BMI, blood pressure, neuroticism and anxiety, glaucoma and retina pathology, lung function and mortality), CXCL17 (a chemokine interacting with CXCL9 that plays a role in tumor genesis, antimicrobial defense through monocytes, macrophages and dendritic cells) and GDF15 (implicated in BMI, liver function, systemic lupus erythematosus and COVID-19). Overall, we found distinct results when using a data-driven approach to model PPIs using interactions from our machine-learning models versus using the most up-to-date experimental biological knowledge from the STRING database.

We further examined the roles and functions of the 20 proteins comprising the ProtAge20 score, which together capture ~95% of the 204-protein model’s ability to predict age. These key APs are involved in (1) cell adhesion and extracellular matrix (ECM) interactions (ELN, COL6A3, CDCP1, PODXL2, LTBP2, SCARF2 and ENG); (2) immune response and inflammation (CXCL17, LECT2, SCARF2 and GDF15); (3) hormone regulation and reproduction (FSHB, AGRP and ACRV1); (4) cell signaling (EDA2R, SCARF2 and PTPRR); (5) protease activity and enzymatic function (KLK3 and KLK7:); (6) regulation of body weight and energy balance (GDF15 and AGRP); (7) neuronal structure and function (GFAP and NEFL); and (8) development and differentiation (EDA2R, LTBP2 and ENG).

### Comparisons with existing DNAm and proteomic age clocks

**Extended Data Fig. 9:**
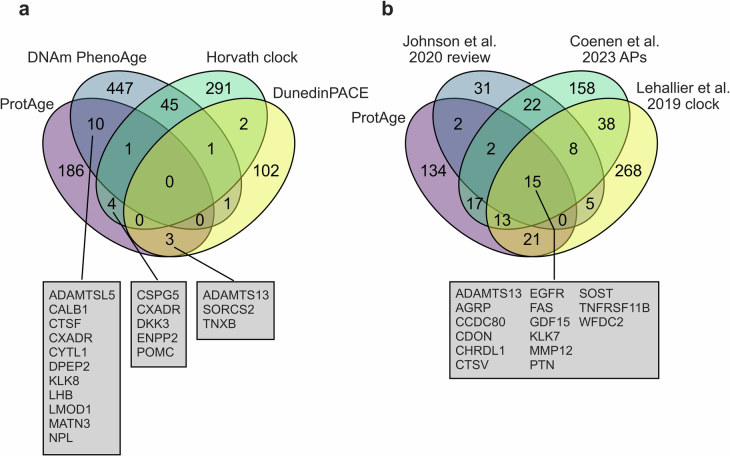
Overlap of ProtAge APs with existing DNAm and proteomic clock publications. **a)** Overlap between genes coding for the 204 ProtAge APs versus genes mapped by proximity to CpGs from common DNAm clocks. **b)** Overlap between 204 ProtAge APs versus a recent systematic review of APs (Johnson et al. 2020), a recent comprehensive analysis of SOMAscan proteins associated with age (Coenen et al. 2023), and a recent proteomic aging clock created using SOMAscan data (Lehallier et al. 2019). APs: aging-related proteins, DNAm: DNA methylation.

Proteins selected by our model showed very little overlap with corresponding genes from leading DNAm clocks, including the Horvath clock^22^, DNAm PhenoAge^23^ and DunedinPACE^24^ (Extended Data Fig. 9a). Five corresponding genes from ProtAge overlapped with genes mapped to CpGs (by proximity) in the Horvath clock (*CSPG5*, *CXADR*, *DKK3*, *ENPP2* and *POMC*) and 11 ProtAge genes overlapped with proximity genes mapped to DNAm PhenoAge (*AMANTSL5*, *CALB1*, *CTSF*, *CXADR*, *CYTL1*, *DPEP2*, *KLK8*, *LHB*, *LMOD1*, *MATN3* and *NPL*). Only three ProtAge genes overlapped with proximity genes mapped to DunedinPACE (*ADAMTS13*, *SORCS2* and *TNXB*).

We also compared our findings with three of the largest published studies on proteomic aging, including (1) a systematic review of studies (*n* = 32) reporting protein associations with age (Johnson et al.^11^), in which the authors developed a proteomic age clock using 85 proteins associated with age in at least three previous studies and validated it in the INTERVAL study (*n* = 3,301); (2) a recent study that identified 273 APs across several cohorts (Coenen et al.^10^) (*n* = 37,650); and (3) a clock consisting of 373 APs developed in the INTERVAL and LonGenity cohorts (Lehallier et al.^12^) (*n* = 4,263).

While the proteins selected by our model showed a greater overlap with those found in existing proteomic clocks, 134 of our ProtAge APs (64%) were not identified in any of these major previous studies on proteomic aging (Extended Data Fig. 9b). Despite representing a largely novel set of APs, ProtAge also includes 15 APs present in the Johnson et al., Coenen et al. and Lehallier et al. analyses (Extended Data Fig. 9b and Extended Data Table 1). Notably, none of these 15 proteins overlap with corresponding genes from any of the DNAm clocks, suggesting that DNAm and proteomic clocks seem to converge on different gene sets. The overlap between the 204 ProtAge APs with all previous studies described here is shown in Supplementary Table 1. Last, the top protein identified in a previously published protein inflammation clock^15^ (CXCL9, an inflammatory cytokine) was identified as an AP in our model, albeit not within the top 20 proteins in our model. None of the ProtAge20 proteins or the top 20 proteins in the full ProtAge model were identified in this previous inflammation clock paper, including several inflammation-related proteins from our ProtAge model (GDF15 and CXCL17).

## Discussion

Our analyses of proteomic data from UK, Chinese and Finnish populations show that proteomic age signatures estimated in the UKB capture information that is highly generalizable across populations of diverse genetic ancestry and diverse levels of morbidity. Our study provides new evidence that proteomic aging is a common feature underlying a large and diverse range of traits related to physical function, frailty and cognitive status, in addition to established aging biomarkers (for example, telomeres and IGF-1). While previous studies have reported that DNAm age is not related to telomere length^25^, we show that proteomic aging is strongly inversely associated with telomere length, a key cellular hallmark of aging^9^. Of note, our study provides comprehensive and well-powered evidence demonstrating that proteomic aging is a reliable predictor of mortality and multimorbidity, and is associated with future risk of all 14 noncancer diseases studied and four common cancers (esophageal, prostate, lung and non-Hodgkin lymphoma). Our study also demonstrates that our proteomic age clock generalizes well to age groups and morbidity profiles beyond those represented in the UKB population. Of particular note is that our proteomic age model performed well in FinnGen participants (age range 20–80 years) who were largely free of disease and mortality and who were up to 20 years younger than participants in the UKB (age range 40–70 years).

This study has directly tested associations of proteomic aging with disease, multimorbidity and age-related functional status in a comprehensive manner within a large and well-powered sample. While one previous study did systematically investigate different biological clocks against 27 major disease outcomes, the proteomic age clock tested was associated with only one disease at nominal significance, likely due to a lack of statistical power in this relatively small study (*n* = 805)^17^. Moreover, we have reduced the impact of reverse causation bias in our study by restricting the analyses to participants without any major lifetime disease diagnoses and demonstrate consistent findings with the overall analyses.

When compared to the limited number of physiological and biological function measures that were analyzed in the previous proteomic clock analyses described above, our ProtAge clock demonstrated improved performance. Coenen et al.^10^ reported only marginally significant partial correlations between their proteomic age clock and blood uric acid and magnesium, but nonsignificant correlations with the other 57 blood markers tested, including albumin, creatinine, γ-glutamyl transferase and C-reactive protein, all of which were strongly associated with ProtAgeGap in our study. The Johnson et al.^11^ clock study did not investigate mortality, morbidity or any frailty or cognitive measures. As these previous papers used SOMAscan proteomics, it is unclear whether their lack of overlap with ProtAge APs and differences in aging phenotype associations may reflect differences between the Olink and SOMAscan platforms. Previous research comparing SOMAscan and Olink data directly demonstrated substantial discrepancies in protein–phenotype associations and genetic protein quantitative trait loci mapping between the two platforms^26^; however, an alternative explanation may be small sample sizes in these previous proteomic age clock studies.

Our ProtAge clock also provides an advantage over many state-of-the-art DNAm clocks. It has been observed that the so-called ‘first generation’ DNAm clocks trained to predict age (for example, the Horvath clock) are only weakly associated with mortality risk and aging-related function^5,7^. To capture stronger associations with morbidity and mortality, newer DNAm clocks were subsequently developed that are not trained on chronological age itself, but rather are trained to predict composite phenotypic age variables that are usually weighted combinations of age and biological markers of morbidity (for example, albumin, creatinine and C-reactive protein)^23,24,27^. An advantage of our proteomic age clock is that it can be constructed by training to predict only age and still remain strongly associated with mortality and morbidity, and that this approach can be still translated to smaller independent samples (CKB and FinnGen) with very different study designs, participant genetic backgrounds and participant morbidity profiles.

One reason that DNAm-based clocks built to predict age may not predict functional and disease outcomes as well is the lack of strong correlation between age-related changes in gene expression (the functional consequence of DNAm) and age-related protein expression. Recent research using kidney and heart tissue in mice^28,29^, in addition to brain tissue from humans and rhesus macaques^30^, demonstrated that age-related changes in messenger RNA and protein levels were not strongly correlated. A key example of highly important proteins in our models whose abundance is not well correlated with mRNA are ECM proteins such as elastin (ELN) and collagens (COL6A3). These proteins have long half-lives that make them particularly susceptible to aging-related degradation and post-translational modifications, which contributes to the structural tissue damage encountered during aging^31^. Elastin fragmentation is a key contributor to vascular aging and is implicated in hypertension and cardiovascular outcomes^32^, although recent research also suggests a role of elastin degradation in CKD^33^ and aging of cerebral arteries^34^. Additionally, functional research in *Caenorhabditis* *elegans* suggests that ECM remodeling is required to promote longevity and that known genetic and pharmacological longevity interventions slow age-related collagen stiffening^35^. Our work provides new evidence from a human population study that ECM dynamics warrant consideration as an emerging hallmark of aging^36^, which may be difficult to capture with DNAm information.

Further, recent research in blood-derived human CD8^+^ T cell populations reported weak correlation between mRNA and protein abundance in immune cells^37^. Given the key role of several immune and inflammation proteins in our ProtAge model (CXCL17, LECT2, SCARF2 and GDF15) and the larger literature demonstrating the importance of immune-related inflammation in aging (inflammaging^38^), this important axis of proteomic aging is also unlikely to be captured via DNAm or transcriptional age clocks.

As 50% of individuals with incident AD in our analysis were younger than 75 years at diagnosis, one of the advantages of our proteomic age clock may be detection or risk prediction of early onset AD. Notably, although recent reviews report that *APOE* (a gene strongly linked to AD risk) and *FOXO3* (a transcription factor involved in apoptosis and DNA repair) are the only two genes whose associations with lifespan and longevity are consistently replicated across studies^2,39^, neither were found to be APs in our study. This highlights a possible disconnect between genetic determinants of lifespan and proteomic signals of aging, although it is also possible that tissue-specific expression of these proteins is not conducive to capturing a blood-based aging signal.

Our analyses also showed consistent associations between ProtAgeGap and four cancers (lung, prostate, esophageal and non-Hodgkin lymphoma) after covariate adjustment. Furthermore, various aspects of cancer development emerged in our analysis of ProtAge protein pathways. Some of the remaining nonassociated cancers had low or insufficient numbers of cases, indicating that we may have lacked statistical power to detect associations. For other cancers with adequate sample size (for example, colorectal cancer), it is possible that the proteomics panel used did not contain the relevant proteins for that cancer or that there is no reliable signature for these proteins in plasma.

Our study approach has many strengths, including our use of gradient-boosting (LightGBM) models that allow for nonlinear associations and account for interactions between all proteins. Further, our model benchmarking process shows that our gradient-boosting model provides substantially greater generalizability for estimating proteomic age in independent data compared to LASSO, elastic net or neural networks; however, our study also had several limitations. First, our model only used the Olink Explore 3072 assay, currently available in the UKB, and therefore did not capture all proteins covered in other platforms and panels, including the larger Olink HT (~5,000 proteins) or SOMAscan (>10,000 proteins) panels. Second, our datasets did not have DNAm data that would allow direct comparisons between proteomic and DNAm age clocks.

In summary, our study provides evidence that plasma proteomics is a powerful tool for measuring biological age and can be used to quantify a biological aging signature that is involved in most common age-related diseases in adult populations. Our work demonstrates that development of proteomic aging clocks can be used as a reliable tool to identify biological mechanisms involved in disease multimorbidity, and may serve as useful tools for identification of protein targets for possible drug treatment or lifestyle modification to reduce premature mortality and reduce or delay the onset of major age-related diseases and multimorbidity.

## Methods

### Study participants

The UKB is a prospective cohort study with extensive genetic and phenotype data available for 502,505 individuals resident in the United Kingdom who were recruited between 2006 and 2010^40^. The full UKB protocol is available online (https://www.ukbiobank.ac.uk/media/gnkeyh2q/study-rationale.pdf). We restricted our UKB sample to those participants with Olink Explore data available at baseline who were randomly sampled from the main UKB population (*n* = 45,441).

The CKB is a prospective cohort study of 512,724 adults aged 30–79 years who were recruited from ten geographically diverse (five rural and five urban) areas across China between 2004 and 2008. Details on the CKB study design and methods have been previously reported^41^. We restricted our CKB sample to those participants with Olink Explore data available at baseline in a nested case–cohort study of IHD and who were genetically unrelated to each other (*n* = 3,977).

The FinnGen study is a public–private partnership research project that has collected and analyzed genome and health data from 500,000 Finnish biobank donors to understand the genetic basis of diseases^42^. FinnGen includes nine Finnish biobanks, research institutes, universities and university hospitals, 13 international pharmaceutical industry partners and the Finnish Biobank Cooperative (FINBB). The project utilizes data from the nationwide longitudinal health register collected since 1969 from every resident in Finland. In FinnGen, we restricted our analyses to those participants with Olink Explore data available and passing proteomic data quality control (*n* = 1,990).

### Proteomic profiling

Proteomic profiling in the UKB, CKB and FinnGen was carried out for protein analytes measured via the Olink Explore 3072 platform that links four Olink panels (Cardiometabolic, Inflammation, Neurology and Oncology). For all cohorts, the preprocessed Olink data were provided in the arbitrary NPX unit on a log_2_ scale. In the UKB, the random subsample of proteomics participants (*n* = 45,441) were selected by removing those in batches 0 and 7. Randomized participants selected for proteomic profiling in the UKB have been shown previously to be highly representative of the wider UKB population^43^. UKB Olink data are provided as Normalized Protein eXpression (NPX) values on a log_2_ scale, with details on sample selection, processing and quality control documented online.

In the CKB, stored baseline plasma samples from participants were retrieved, thawed and subaliquoted into multiple aliquots, with one (100 µl) aliquot used to make two sets of 96-well plates (40 µl per well). Both sets of plates were shipped on dry ice, one to the Olink Bioscience Laboratory at Uppsala (batch one, 1,463 unique proteins) and the other shipped to the Olink Laboratory in Boston (batch two, 1,460 unique proteins), for proteomic analysis using a multiplex proximity extension assay, with each batch covering all 3,977 samples. Samples were plated in the order they were retrieved from long-term storage at the Wolfson Laboratory in Oxford and normalized using both an internal control (extension control) and an inter-plate control and then transformed using a predetermined correction factor. The limit of detection (LOD) was determined using negative control samples (buffer without antigen). A sample was flagged as having a quality control warning if the incubation control deviated more than a predetermined value (±0.3) from the median value of all samples on the plate (but values below LOD were included in the analyses).

In the FinnGen study, blood samples were collected from healthy individuals and EDTA-plasma aliquots (230 µl) were processed and stored at −80 °C within 4 h. Plasma aliquots were subsequently thawed and plated in 96-well plates (120 µl per well) as per Olink’s instructions. Samples were shipped on dry ice to the Olink Bioscience Laboratory (Uppsala) for proteomic analysis using the 3,072 multiplex proximity extension assay. Samples were sent in three batches and to minimize any batch effects, bridging samples were added according to Olink’s recommendations. In addition, plates were normalized using both an internal control (extension control) and an inter-plate control and then transformed using a predetermined correction factor. The LOD was determined using negative control samples (buffer without antigen). A sample was flagged as having a quality control warning if the incubation control deviated more than a predetermined value (±0.3) from the median value of all samples on the plate (but values below LOD were included in the analyses).

We excluded from analysis any proteins not available in all three cohorts, as well as an additional three proteins that were missing in over 10% of the UKB sample (CTSS, PCOLCE and NPM1), leaving a total of 2,897 proteins for analysis. After missing data imputation (see below), proteomic data were normalized separately within each cohort by first rescaling values to be between 0 and 1 using MinMaxScaler() from scikit-learn and then centering on the median.

### Outcomes

UKB aging biomarkers were measured using baseline nonfasting blood serum samples as previously described^44^. Biomarkers were previously adjusted for technical variation by the UKB, with sample processing (https://biobank.ndph.ox.ac.uk/showcase/showcase/docs/serum_biochemistry.pdf) and quality control (https://biobank.ndph.ox.ac.uk/showcase/ukb/docs/biomarker_issues.pdf) procedures described on the UKB website. Field IDs for all biomarkers and measures of physical and cognitive function are shown in Supplementary Table 18. Poor self-rated health, slow walking pace, self-rated facial aging, feeling tired/lethargic every day and frequent insomnia were all binary dummy variables coded as all other responses versus responses for ‘Poor’ (overall health rating; field ID 2178), ‘Slow pace’ (usual walking pace; field ID 924), ‘Older than you are’ (facial aging; field ID 1757), ‘Nearly every day’ (frequency of tiredness/lethargy in last 2 weeks; field ID 2080) and ‘Usually’ (sleeplessness/insomnia; field ID 1200), respectively. Sleeping 10+ hours per day was coded as a binary variable using the continuous measure of self-reported sleep duration (field ID 160). Systolic and diastolic blood pressure were averaged across both automated readings. Standardized lung function (FEV_1_) was calculated by dividing the FEV_1_ best measure (field ID 20150) by standing height squared (field ID 50). Hand grip strength variables (field ID 46,47) were divided by weight (field ID 21002) to normalize according to body mass. Frailty index was calculated using the algorithm previously developed for UKB data by Williams et al.^21^. Components of the frailty index are shown in Supplementary Table 19. Leukocyte telomere length was measured as the ratio of telomere repeat copy number (T) relative to that of a single copy gene (S; *HBB*, which encodes human hemoglobin subunit β)^45^. This T:S ratio was adjusted for technical variation and then both log-transformed and z-standardized using the distribution of all individuals with a telomere length measurement.

Detailed information about the linkage procedure (https://biobank.ctsu.ox.ac.uk/crystal/refer.cgi?id=115559) with national registries for mortality and cause of death information in the UKB is available online. Mortality data were accessed from the UKB data portal on 23 May 2023, with a censoring date of 30 November 2022 for all participants (12–16 years of follow-up).

Data used to define prevalent and incident chronic diseases in the UKB are outlined in Supplementary Table 20. In the UKB, incident cancer diagnoses were ascertained using International Classification of Diseases (ICD) diagnosis codes and corresponding dates of diagnosis from linked cancer and mortality register data. Incident diagnoses for all other diseases were ascertained using ICD diagnosis codes and corresponding dates of diagnosis taken from linked hospital inpatient, primary care and death register data. Primary care read codes were converted to corresponding ICD diagnosis codes using the lookup table provided by the UKB. Linked hospital inpatient, primary care and cancer register data were accessed from the UKB data portal on 23 May 2023, with a censoring date of 31 October 2022; 31 July 2021 or 28 February 2018 for participants recruited in England, Scotland or Wales, respectively (8–16 years of follow-up).

In the CKB, information about incident disease and cause-specific mortality was obtained by electronic linkage, via the unique national identification number, to established local mortality (cause-specific) and morbidity (for stroke, IHD, cancer and diabetes) registries and to the health insurance system that records any hospitalization episodes and procedures^41,46^. All disease diagnoses were coded using the ICD-10, blinded to any baseline information, and participants were followed up to death, loss-to-follow-up or 1 January 2019. ICD-10 codes used to define diseases studied in the CKB are shown in Supplementary Table 21.

### Missing data imputation

Missing values for all nonproteomics UKB data were imputed using the R package missRanger^47^, which combines random forest imputation with predictive mean matching. We imputed a single dataset using a maximum of ten iterations and 200 trees. All other random forest hyperparameters were left at default values. The imputation dataset included all baseline variables available in the UKB as predictors for imputation, excluding variables with any nested response patterns. Responses of ‘do not know’ were set to ‘NA’ and imputed. Responses of ‘prefer not to answer’ were not imputed and set to NA in the final analysis dataset. Age and incident health outcomes were not imputed in the UKB. CKB data had no missing values to impute.

Protein expression values were imputed in the UKB and FinnGen cohort using the miceforest package in Python. All proteins except those missing in >30% of participants were used as predictors for imputation of each protein. We imputed a single dataset using a maximum of five iterations. All other parameters were left at default values.

### Calculation of chronological age measures

In the UKB, age at recruitment (field ID 21022) is only provided as a whole integer value. We derived a more accurate estimate by taking month of birth (field ID 52) and year of birth (field ID 34) and creating an approximate date of birth for each participant as the first day of their birth month and year. Age at recruitment as a decimal value was then calculated as the number of days between each participant’s recruitment date (field ID 53) and approximate birth date divided by 365.25. Age at the first imaging follow-up (2014+) and the repeat imaging follow-up (2019+) were then calculated by taking the number of days between the date of each participant’s follow-up visit and their initial recruitment date divided by 365.25 and adding this to age at recruitment as a decimal value. Recruitment age in the CKB is already provided as a decimal value.

### Model benchmarking

We compared the performance of six different machine-learning models (LASSO, elastic net, LightGBM and three neural network architectures: multilayer perceptron, a residual feedforward network (ResNet) and a retrieval-augmented neural network for tabular data (TabR)) for using plasma proteomic data to predict age. For each model, we trained a regression model using all 2,897 Olink protein expression variables as input to predict chronological age. All models were trained using fivefold cross-validation in the UKB training data (*n* = 31,808) and were tested against the UKB holdout test set (*n* = 13,633), as well as independent validation sets from the CKB and FinnGen cohorts. We found that LightGBM provided the second-best model accuracy among the UKB test set, but showed markedly better performance in the independent validation sets (Supplementary Fig. 1).

LASSO and elastic net models were calculated using the scikit-learn package in Python. For the LASSO model, we tuned the alpha parameter using the LassoCV function and an alpha parameter space of [1 × 10^−15^, 1 × 10^−10^, 1 × 10^−8^, 1 × 10^−5^, 1 × 10^−4^, 1 × 10^−3^, 1 × 10^−2^, 1, 5, 10, 50 and 100]. Elastic net models were tuned for both alpha (using the same parameter space) and L1 ratio drawn from the following possible values: [0.1, 0.5, 0.7, 0.9, 0.95, 0.99 and 1].

The LightGBM model hyperparameters were tuned via fivefold cross-validation using the Optuna module in Python^48^, with parameters tested across 200 trials and optimized to maximize the average *R*^2^ of the models across all folds.

The neural network architectures tested in this analysis were selected from a list of architectures that performed well on a variety of tabular datasets. The architectures considered were (1) a multilayer perceptron; (2) ResNet; and (3) TabR. All neural network model hyperparameters were tuned via fivefold cross-validation using Optuna across 100 trials and optimized to maximize the average *R*^2^ of the models across all folds.

### Calculation of ProtAge

Using gradient boosting (LightGBM) as our selected model type, we initially ran models trained separately on males and females; however, the male- and female-only models showed similar age prediction performance to a model with both sexes (Supplementary Fig. 8a–c) and protein-predicted age from the sex-specific models were nearly perfectly correlated with protein-predicted age from the model using both sexes (Supplementary Fig. 8d,e). We further found that when looking at the most important proteins in each sex-specific model, there was a large consistency across males and females. Specifically, 11 of the top 20 most important proteins for predicting age according to SHAP values were shared across males and females and all 11 shared proteins showed consistent directions of effect for males and females (Supplementary Fig. 9a,b; ELN, EDA2R, LTBP2, NEFL, CXCL17, SCARF2, CDCP1, GFAP, GDF15, PODXL2 and PTPRR). We therefore calculated our proteomic age clock in both sexes combined to improve the generalizability of the findings.

To calculate proteomic age, we first split all UKB participants (*n* = 45,441) into 70:30 train–test splits. In the training data (*n* = 31,808), we trained a model to predict age at recruitment using all 2,897 proteins in a single LightGBM^18^ model. First, model hyperparameters were tuned via fivefold cross-validation using the Optuna module in Python^48^, with parameters tested across 200 trials and optimized to maximize the average *R*^2^ of the models across all folds. We then carried out Boruta feature selection via the SHAP-hypetune module. Boruta feature selection works by making random permutations of all features in the model (called shadow features), which are essentially random noise^19^. In our use of Boruta, at each iterative step these shadow features were generated and a model was run with all features and all shadow features. We then removed all features that did not have a mean of the absolute SHAP value that was higher than all random shadow features. The selection processes ended when there were no features remaining that did not perform better than all shadow features. This procedure identifies all features relevant to the outcome that have a greater influence on prediction than random noise. When running Boruta, we used 200 trials and a threshold of 100% to compare shadow and real features (meaning that a real feature is selected if it performs better than 100% of shadow features). Third, we re-tuned model hyperparameters for a new model with the subset of selected proteins using the same procedure as before. Both tuned LightGBM models before and after feature selection were checked for overfitting and validated by performing fivefold cross-validation in the combined train set and testing the performance of the model against the holdout UKB test set. Across all analysis steps, LightGBM models were run with 5,000 estimators, 20 early stopping rounds and using *R*^2^ as a custom evaluation metric to identify the model that explained the maximum variation in age (according to *R*^2^).

Once the final model with Boruta-selected APs was trained in the UKB, we calculated protein-predicted age (ProtAge) for the entire UKB cohort (*n* = 45,441) using fivefold cross-validation. Within each fold, a LightGBM model was trained using the final hyperparameters and predicted age values were generated for the test set of that fold. We then combined the predicted age values from each of the folds to create a measure of ProtAge for the entire sample. ProtAge was calculated in the CKB and FinnGen by using the trained UKB model to predict values in those datasets. Finally, we calculated proteomic aging gap (ProtAgeGap) separately in each cohort by taking the difference of ProtAge minus chronological age at recruitment separately in each cohort.

### Recursive feature elimination using SHAP

For our recursive feature elimination analysis, we started from the 204 Boruta-selected proteins. In each step, we trained a model using fivefold cross-validation in the UKB training data and then within each fold calculated the model *R*^2^ and the contribution of each protein to the model as the mean of the absolute SHAP values across all participants for that protein. *R*^2^ values were averaged across all five folds for each model. We then removed the protein with the smallest mean of the absolute SHAP values across the folds and computed a new model, eliminating features recursively using this method until we reached a model with only five proteins. If at any step of this process a different protein was identified as the least important in the different cross-validation folds, we chose the protein ranked the lowest across the greatest number of folds to remove. We identified 20 proteins as the smallest number of proteins that provide adequate prediction of chronological age, as fewer than 20 proteins resulted in a dramatic drop in model performance (Supplementary Fig. 3d). We re-tuned hyperparameters for this 20-protein model (ProtAge20) using Optuna according to the methods described above, and we also calculated the proteomic age gap according to these top 20 proteins (ProtAgeGap20) using fivefold cross-validation in the entire UKB cohort (*n* = 45,441) using the methods described above.

### Statistical analysis

All statistical analyses were carried out using Python v.3.6 and R v.4.2.2. All associations between ProtAgeGap and aging biomarkers and physical/cognitive function measures in the UKB were tested using linear/logistic regression using the statsmodels module^49^. All models were adjusted for age, sex, Townsend deprivation index, assessment center, self-reported ethnicity (Black, white, Asian, mixed and other), IPAQ activity group (low, moderate and high) and smoking status (never, previous and current). *P* values were corrected for multiple comparisons via the FDR using the Benjamini–Hochberg method^50^.

All associations between ProtAgeGap and incident outcomes (mortality and 26 diseases) were tested using Cox proportional hazards models using the lifelines module^51^. Survival outcomes were defined using follow-up time to event and the binary incident event indicator. For all incident disease outcomes, prevalent cases were excluded from the dataset before models were run. For all incident outcome Cox modeling in the UKB, three successive models were tested with increasing numbers of covariates. Model 1 included adjustment for age at recruitment and sex. Model 2 included all model 1 covariates, plus Townsend deprivation index (field ID 22189), assessment center (field ID 54), physical activity (IPAQ activity group; field ID 22032) and smoking status (field ID 20116). Model 3 included all model 3 covariates plus BMI (field ID 21001) and prevalent hypertension (defined in Supplementary Table 20). *P* values were corrected for multiple comparisons via FDR.

Functional enrichments (GO biological processes, GO molecular function, KEGG and Reactome) and PPI networks were downloaded from STRING (v.12) using the STRING API in Python. For functional enrichment analyses, we used all proteins included in the Olink Explore 3072 platform as the statistical background (except for 19 Olink proteins that could not be mapped to STRING IDs. None of the proteins that could not be mapped were included in our final Boruta-selected proteins). We only considered PPIs from STRING at a high level of confidence (>0.7) from the coexpression data.

SHAP interaction values from the trained LightGBM ProtAge model were retrieved using the SHAP module^20,52^. SHAP-based PPI networks were generated by first taking the mean of the absolute value of each protein–protein SHAP interaction score across all samples. We then used an interaction threshold of 0.0083 and removed all interactions below this threshold, which yielded a subset of variables similar in number to the node degree >2 threshold used for the STRING PPI network. Both SHAP-based and STRING^53^-based PPI networks were visualized and plotted using the NetworkX module^54^.

Cumulative incidence curves and survival tables for deciles of ProtAgeGap were calculated using KaplanMeierFitter from the lifelines module. As our data were right-censored, we plotted cumulative events against age at recruitment on the *x* axis. All plots were generated using matplotlib^55^ and seaborn^56^. The total fold risk of disease according to the top and bottom 5% of the ProtAgeGap was calculated by raising the HR for the disease by the total number of years comparison (12.3 years average ProtAgeGap difference between the top versus bottom 5% and 6.3 years average ProtAgeGap between the top 5% versus those with 0 years of ProtAgeGap).

### Ethics approval

UKB data use (project application no. 61054) was approved by the UKB according to their established access procedures. UKB has approval from the North West Multi-centre Research Ethics Committee as a research tissue bank and as such researchers using UKB data do not require separate ethical clearance and can operate under the research tissue bank approval. The CKB complies with all the required ethical standards for medical research on human participants. Ethical approvals were granted and have been maintained by the relevant institutional ethical research committees in the United Kingdom and China. Study participants in FinnGen provided informed consent for biobank research, based on the Finnish Biobank Act. The FinnGen study is approved by the Finnish Institute for Health and Welfare (permit nos. THL/2031/6.02.00/2017, THL/1101/5.05.00/2017, THL/341/6.02.00/2018, THL/2222/6.02.00/2018, THL/283/6.02.00/2019, THL/1721/5.05.00/2019 and THL/1524/5.05.00/2020), Digital and Population Data Service Agency (permit nos. VRK43431/2017-3, VRK/6909/2018-3 and VRK/4415/2019-3), the Social Insurance Institution (permit nos. KELA 58/522/2017, KELA 131/522/2018, KELA 70/522/2019, KELA 98/522/2019, KELA 134/522/2019, KELA 138/522/2019, KELA 2/522/2020 and KELA 16/522/2020), Findata (permit nos. THL/2364/14.02/2020, THL/4055/14.06.00/2020, THL/3433/14.06.00/2020, THL/4432/14.06/2020, THL/5189/14.06/2020, THL/5894/14.06.00/2020, THL/6619/14.06.00/2020, THL/209/14.06.00/2021, THL/688/14.06.00/2021, THL/1284/14.06.00/2021, THL/1965/14.06.00/2021, THL/5546/14.02.00/2020, THL/2658/14.06.00/2021 and THL/4235/14.06.00/2021), Statistics Finland (permit nos. TK-53-1041-17 and TK/143/07.03.00/2020 (previously TK-53-90-20) TK/1735/07.03.00/2021 and TK/3112/07.03.00/2021) and Finnish Registry for Kidney Diseases permission/extract from the meeting minutes on 4 July 2019.

### Reporting summary

Further information on research design is available in the Nature Portfolio Reporting Summary linked to this article.

## Online content

Any methods, additional references, Nature Portfolio reporting summaries, source data, extended data, supplementary information, acknowledgements, peer review information; details of author contributions and competing interests; and statements of data and code availability are available at 10.1038/s41591-024-03164-7.

## Supplementary information

Supplementary Information Supplementary Figs. 1–9 and supplementary table descriptions.

Reporting Summary

Supplementary Tables 1–21.

## Figures and Tables

**Extended Data Table 1:**
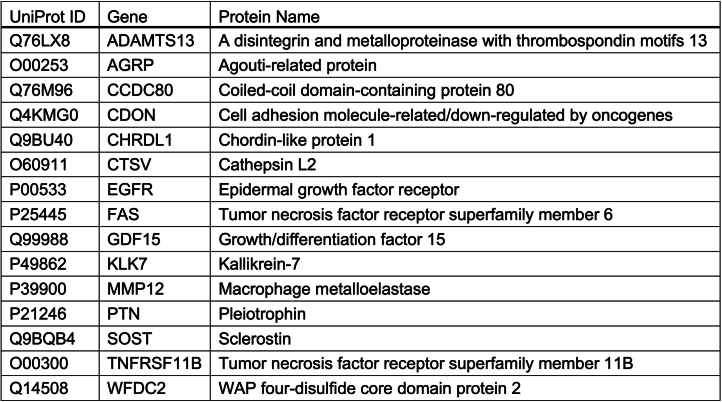
ProtAge APs also associated with age in other major publications

Proteins listed are those also reported to associate with age in Johnson et al. (2020), Coenen et al. (2023), and Lehallier et al. (2019). APs: aging-related proteins.

## Data Availability

UKB data are available through a procedure described at https://www.ukbiobank.ac.uk/enable-your-research. The CKB is a global resource for the investigation of lifestyle, environmental, blood biochemical and genetic factors as determinants of common diseases. The CKB study group is committed to making the cohort data available to the scientific community in China, the United Kingdom and worldwide to advance knowledge about the causes, prevention and treatment of disease. For detailed information on what data are currently available to open-access users and how to apply for them, please visit https://www.ckbiobank.org/data-access. A research proposal will be requested to ensure that any analysis is performed by bona fide researchers. Researchers who are interested in obtaining additional information or data that underline this paper should contact ckbaccess@ndph.ox.ac.uk. For any data that are not currently available via open access, researchers may need to develop a formal collaboration with the CKB study group. FinnGen data can be accessed through Fingenious services (https://site.fingenious.fi/en/) managed by FINBB. Finnish Health Register data can be applied for from Findata (https://findata.fi/en/data/). Experimental protein–protein interaction information used from the STRING database (v.12) can be accessed programmatically using the STRING API (https://string-db.org/) or can be downloaded directly from the STRING website (https://string-db.org/cgi/download.pl).

## Code availability

R and Python code used for data preparation and analysis can be found at https://github.com/miargentieri/proteomic-age-ukb.
