# Supplementary material for: Proteomic aging clock predicts mortality and risk of common age-related diseases in diverse populations
Source: Nat Med. 2024 Aug 8;30(9):2450–60. doi: 10.1038/s41591-024-03164-7 (PMC11405266; doi:10.1038/s41591-024-03164-7)
Supplement: Supplementary file 1 — Supplementary Figs. 1–9 and supplementary table descriptions. [file 41591_2024_3164_MOESM1_ESM.pdf]

nature medicine

Supplementary information https://doi.org/10.1038/s41591-024-03164-7

Proteomic aging clock predicts mortality and risk of common age-related diseases in diverse populations

In the format provided by the authors and unedited

Supplementary Figures

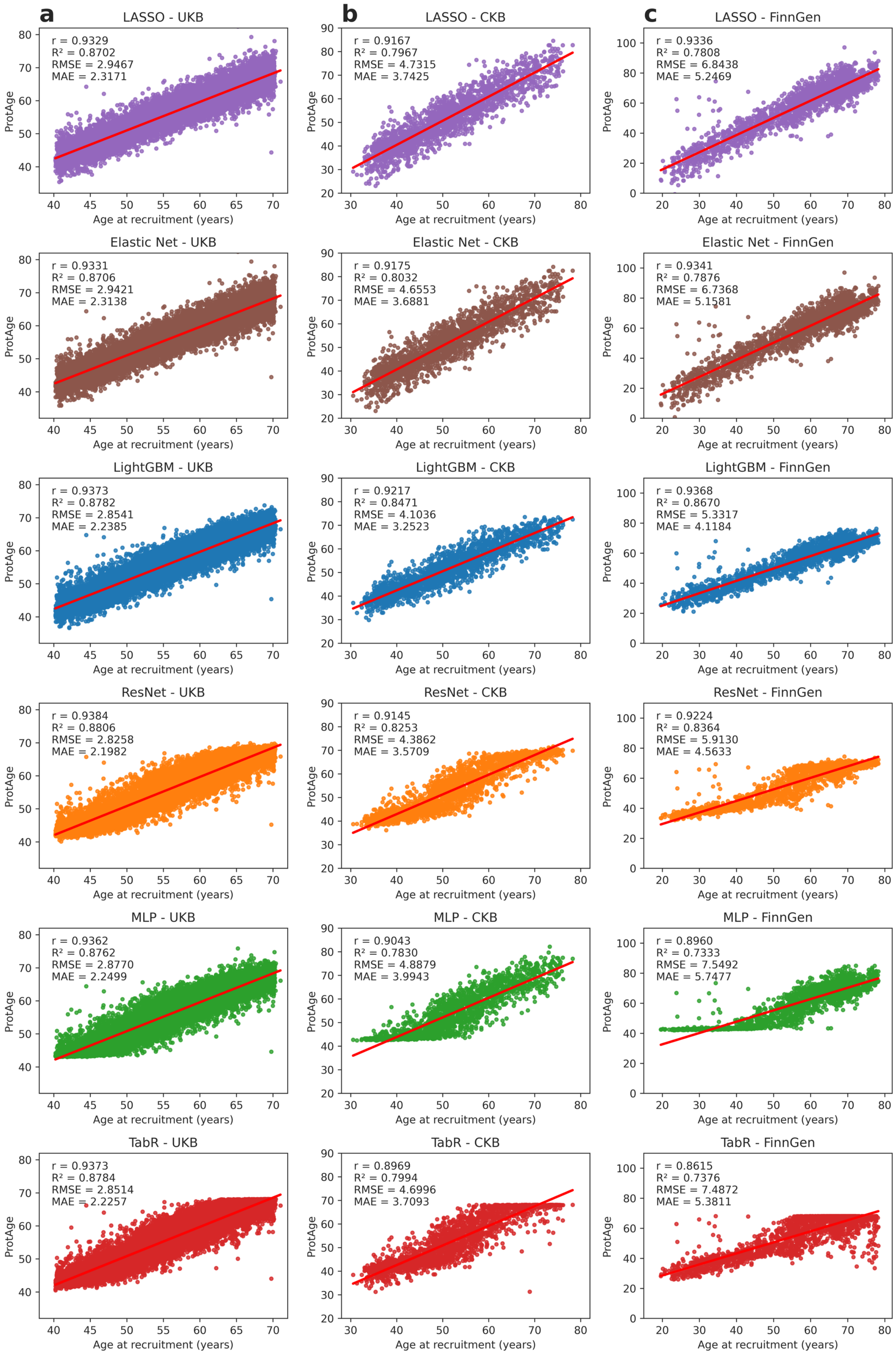

Fig. S1. Model benchmarking for estimation of proteomic age in the UK Biobank and China Kadoorie Biobank. Scatterplots comparing actual chronological age (x-axis) versus protein predicted age (protAge; y-axis) in **a)** the UK Biobank test set (n=13,633); **b)** China Kadoorie Biobank (n=3,977); and **c)** FinnGen (n=1,990). Models compared included two penalized linear regression models (LASSO, elastic net), one gradient boosting machine learning model (LightGBM), and three neural network architectures (ResNet, MLP, TabR). LASSO: least absolute shrinkage and selection operator; MAE: mean absolute error; MLP: multilayer perceptron; RMSE: root mean square error.

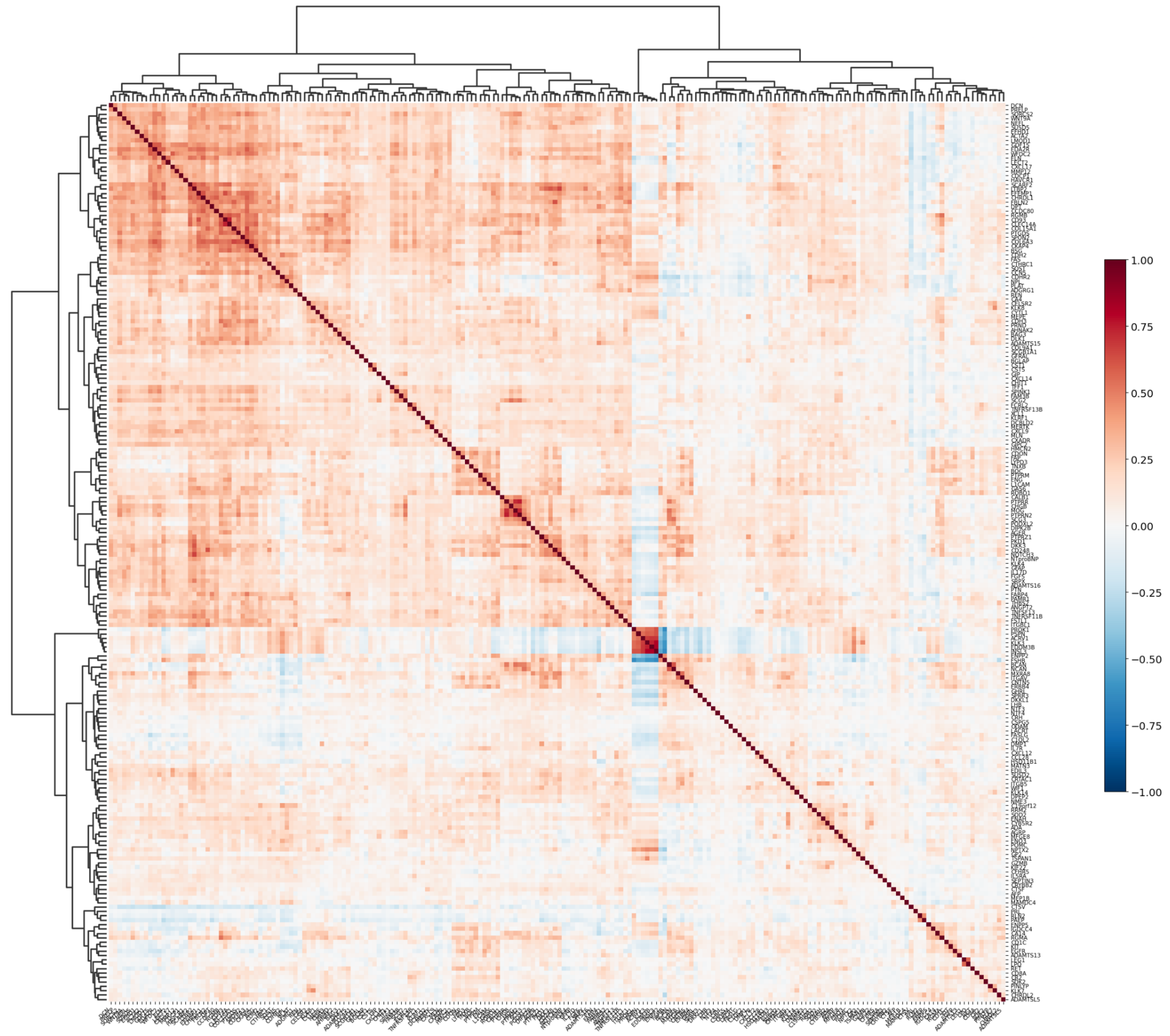

Fig. S2. Correlation structure among the 204 APs in the proteomic age model. Pairwise correlation between each of the 204 APs identified in our work is shown here. Correlation was calculated using Pearson correlation and clustering of protein correlation is calculated using Euclidean distance.

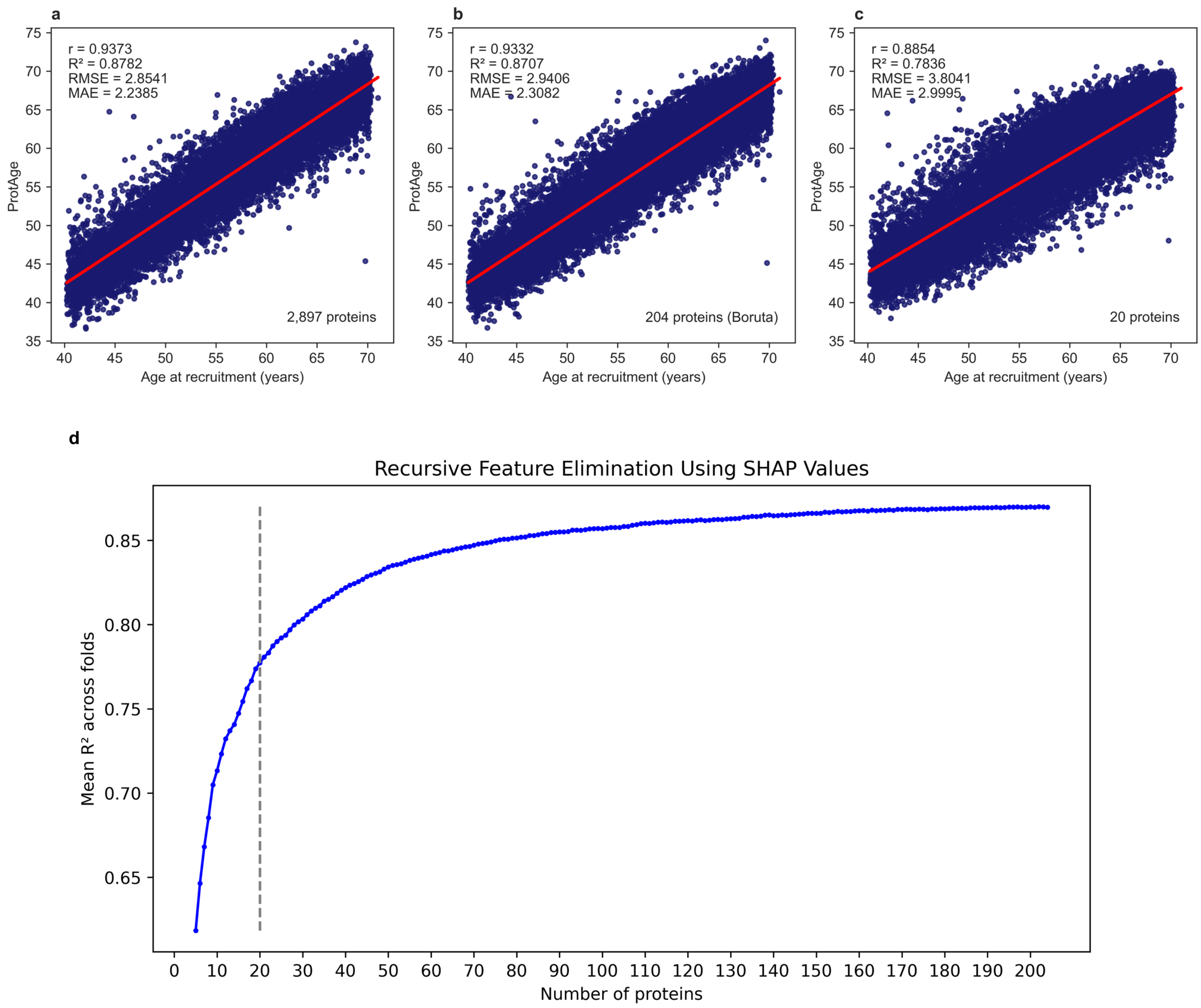

Fig. S3. Performance of proteomic age clocks with decreasing numbers of proteins. Plots shown are the comparison of actual chronological age versus protein predicted age from three LightGBM models using: **a)** all 2,987 proteins considered, **b)** 204 proteins identified in our Boruta feature selection process, **c)** 20 proteins identified through further recursive feature elimination analysis using SHAP values. **d)** Models were tested iteratively using 5-fold cross-validation starting from 204 proteins down to 5 proteins. At each step, the protein with the smallest absolute mean SHAP values across the folds was discarded. For each model, the R^2 of explained variance in chronological age is presented as the average R^2 across all 5 folds. Correlation coefficients (r) shown are from a Pearson correlation test. MAE: mean absolute error; ProtAge: protein predicted age; RMSE: root mean square error.

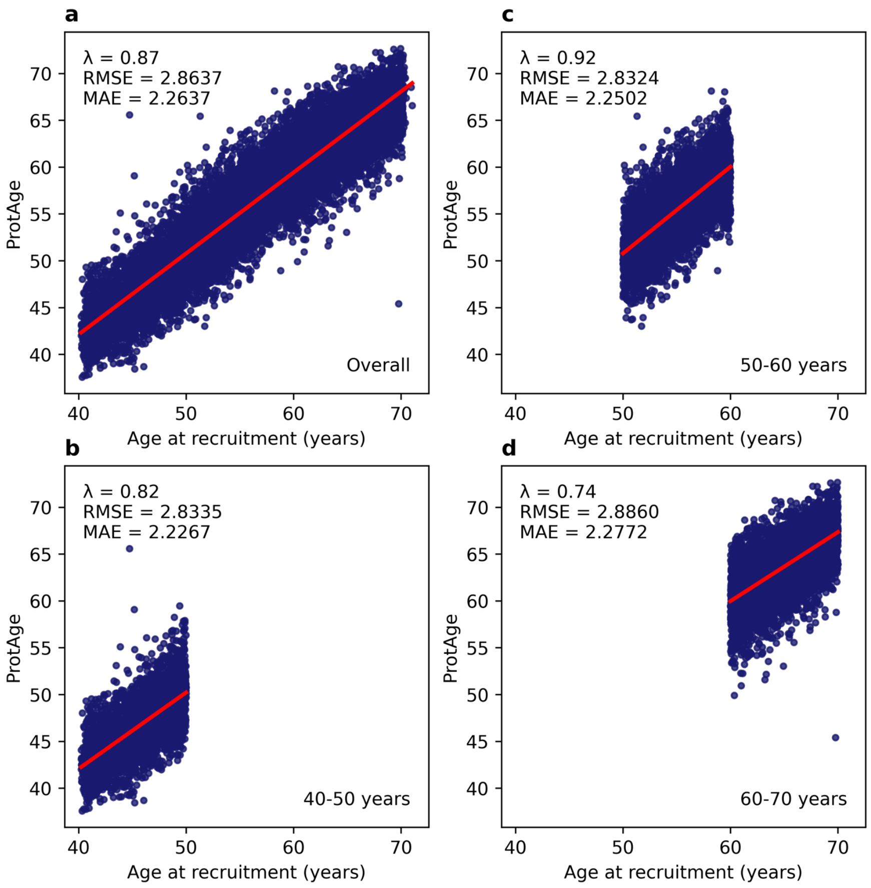

Fig. S4. Proteomic age model performance across age bins in the UKB test set. The performance of our 2,897-protein model is shown in the **a)** full UKB test set (n=13,242), as well as **b)** in the subset of participants aged 40-50 years (n=2,971), **c)** 50-60 years (n= 4,274), and **d)** 60-70 years (n= 5,907). MAE: mean absolute error; RMSE: root mean square error; UKB: UK Biobank.

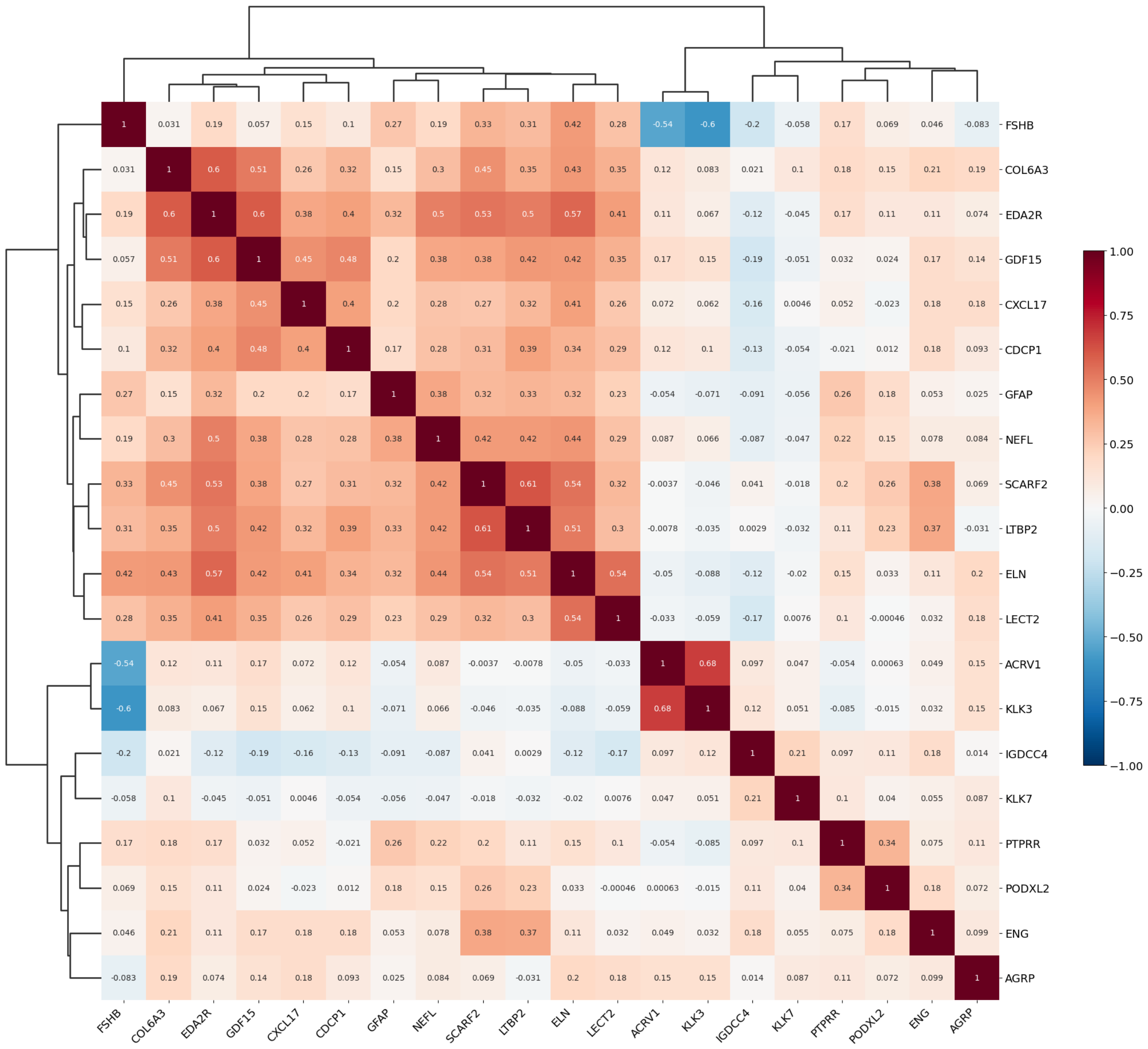

Fig. S5. Correlation structure among the 20 APs in the ProtAge20 model. Pairwise correlation between each of the 20 APs identified in our recursive feature selection is shown here. Correlation was calculated using Pearson correlation and clustering of protein correlation is calculated using Euclidean distance.

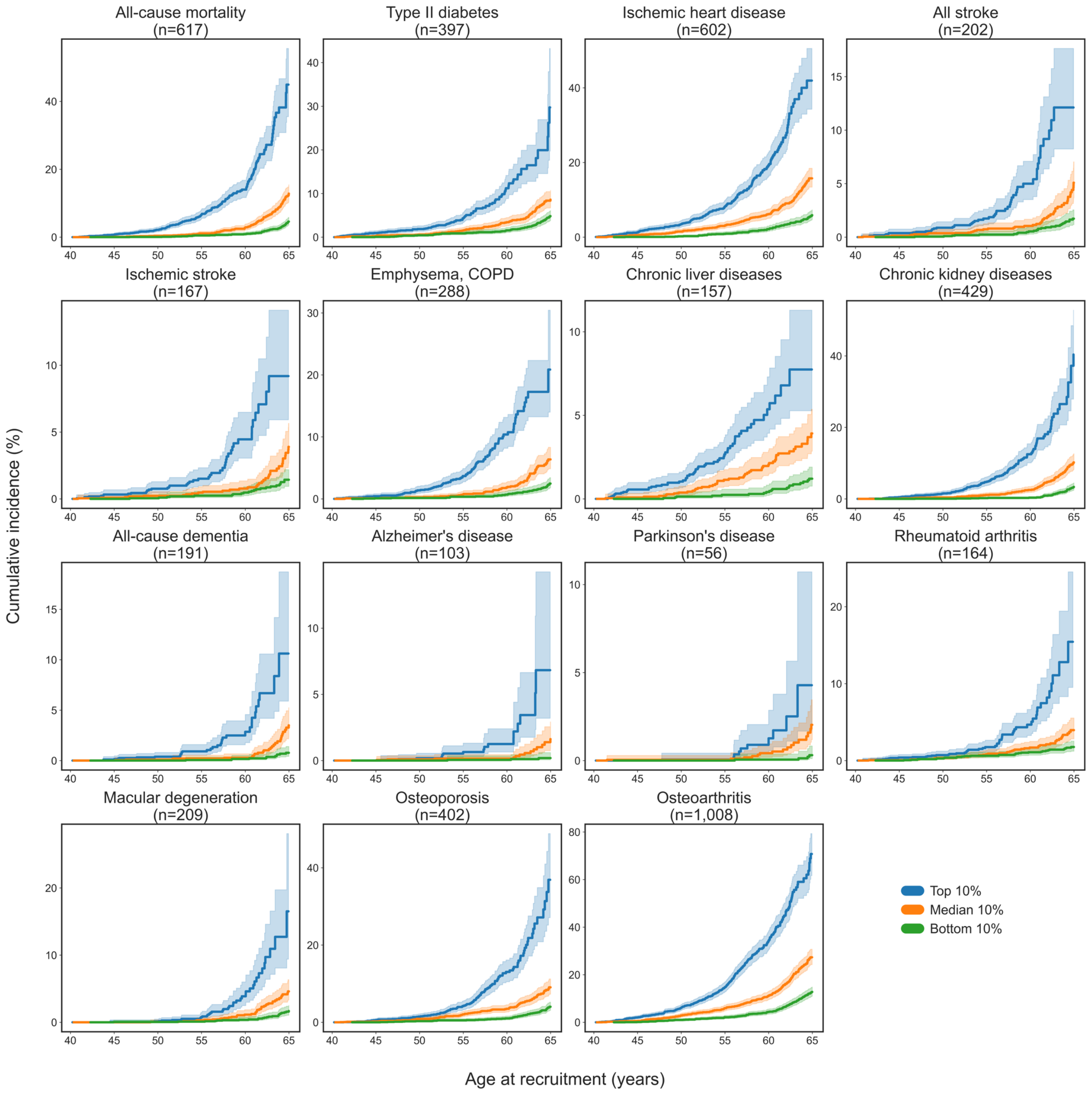

Fig. S6. ProtAgeGap stratifies individuals into divergent age-specific mortality and disease risk trajectories (females only). Cumulative incidence plots for the top, median, and bottom deciles of ProtAgeGap among UK Biobank females (n=24,579). Number of incident cases are shown for each disease – these numbers reflect the total number of incident cases present only among those in the 3 deciles shown, not the full dataset. Incidence rates are shown for the subssequent 11-16 years of follow-up after recruitment for each given age at recruitment (e.g., the cumulative incidence rate shown at age 65 is the rate of incident cases in the 11-16 years of follow up in those aged 65 years at recruitment). All plots show the cumulative density of events at a given timepoint based on the Kaplan-Meier survival function, with 95% confidence intervals in lighter shading. ProtAgeGap: proteomic age gap (in years).

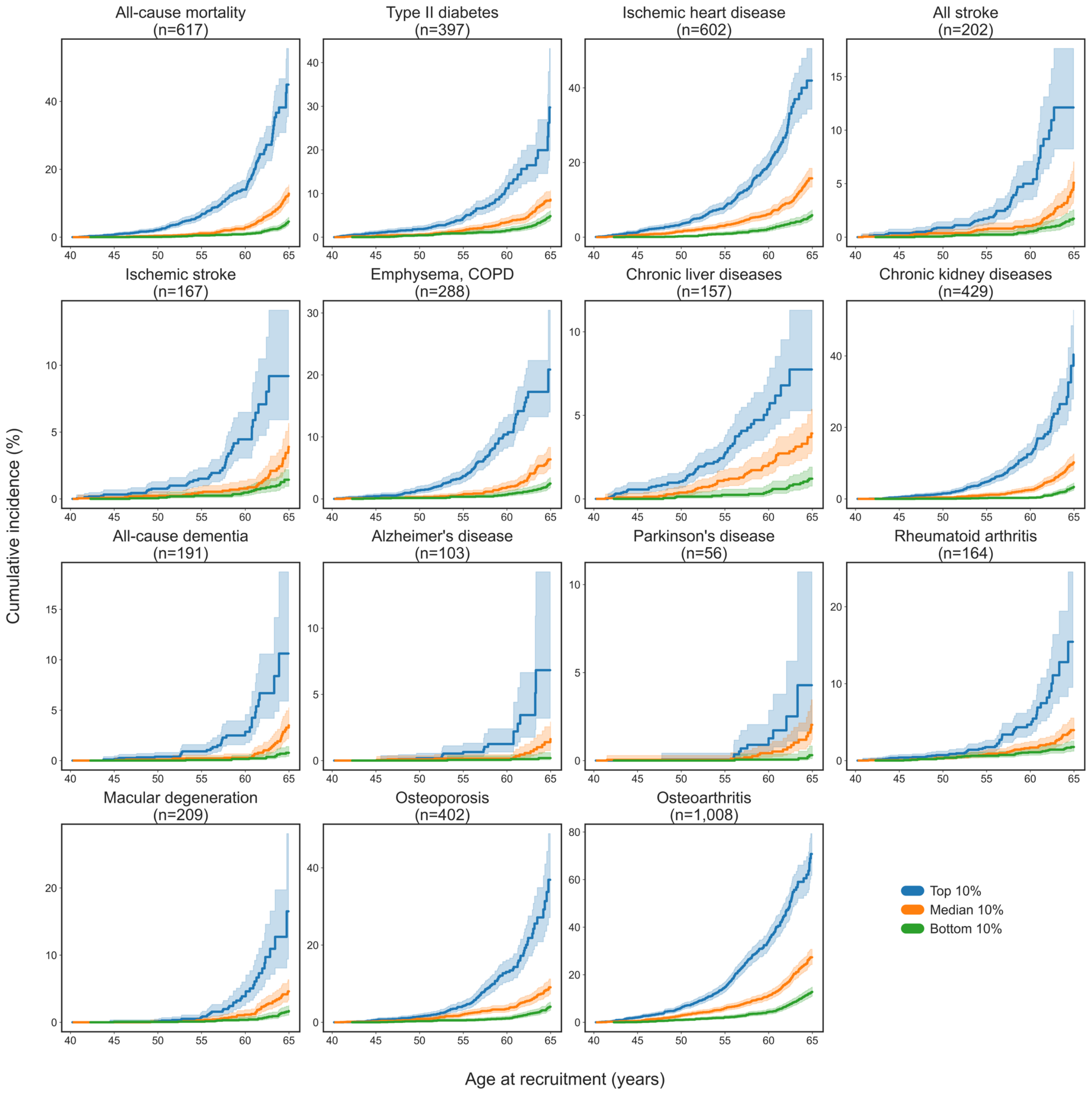

Fig. S7. ProtAgeGap stratifies individuals into divergent age-specific mortality and disease risk trajectories (males only). Cumulative incidence plots for the top, median, and bottom deciles of ProtAgeGap among UK Biobank males (n=20,862). Number of incident cases are shown for each disease – these numbers reflect the total number of incident cases present only among those in the 3 deciles shown, not the full dataset. Incidence rates are shown for the subssequent 11-16 years of follow-up after recruitment for each given age at recruitment (e.g., the cumulative incidence rate shown at age 65 is the rate of incident cases in the 11-16 years of follow up in those aged 65 years at recruitment). All plots show the cumulative density of events at a given timepoint based on the Kaplan-Meier survival function, with 95% confidence intervals in lighter shading. ProtAgeGap: proteomic age gap (in years).

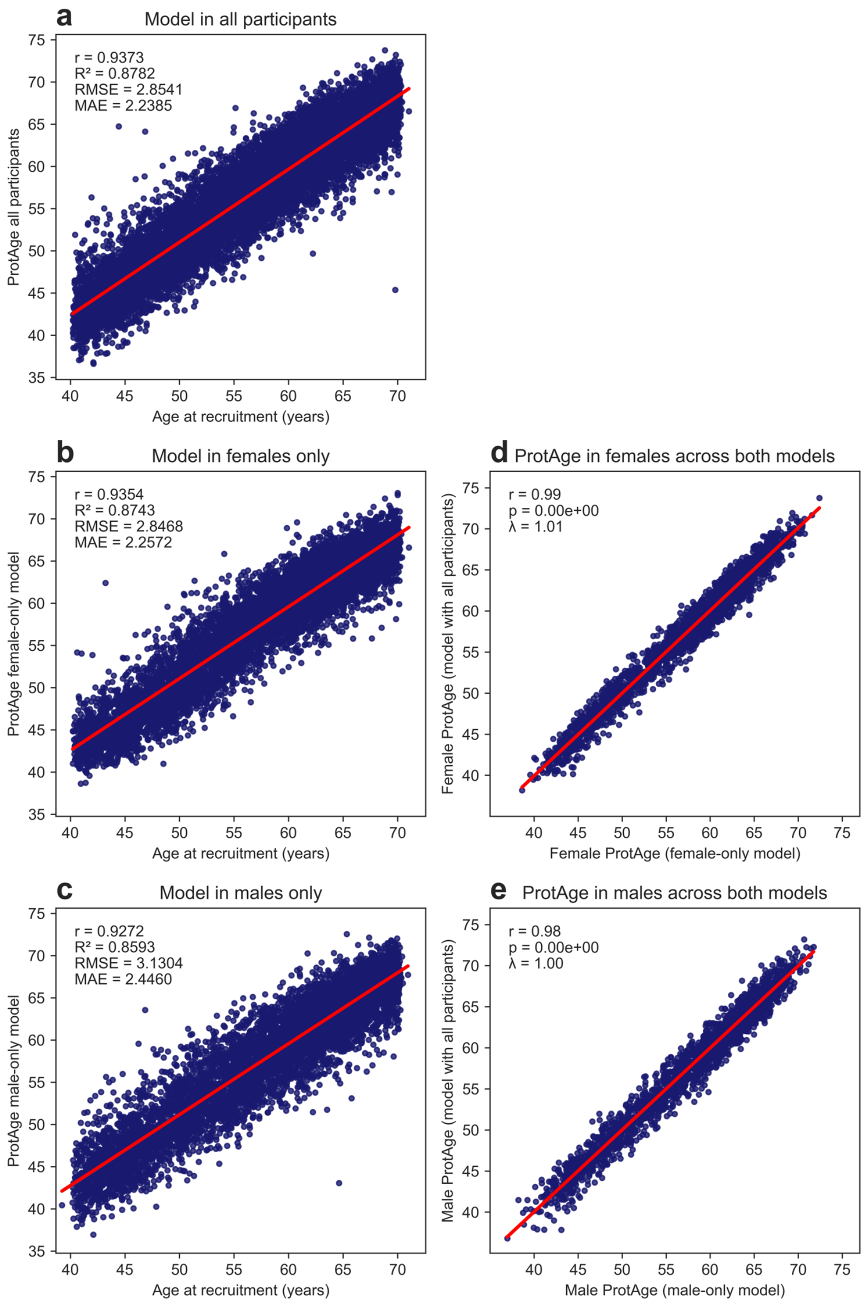

Fig. S8. Proteomic age estimation accuracy by sex. Comparison of actual chronological age versus protein predicted age (ProtAge) for a model using: **a)** all participants (n=13,633), **b)** female participants only (n=7,374), **c)** male participants only (n=6,259). Model accuracy metrics comparing predicted versus actual age values are shown as Pearson r correlation coefficient, R^2, root mean square error (RMSE), and mean absolute error (MAE). **d)** Comparison of protein predicted age (protAge) for the same female participants from the all participant model (y-axis) and model with only female participants (x-axis). **e)** Comparison of protein predicted age (protAge) for the same male participants from the all participant model (y-axis) and model with only male participants (x-axis). In both **d** and **e**, the Pearson r correlation coefficient, p-value for correlation, and slope of the best fit line (λ; calculated using two-sided linear least-squares regression) are shown for comparison of the two predicted ages. P-values are listed as 0 because the number was too small and was rounded to 0 in Python.

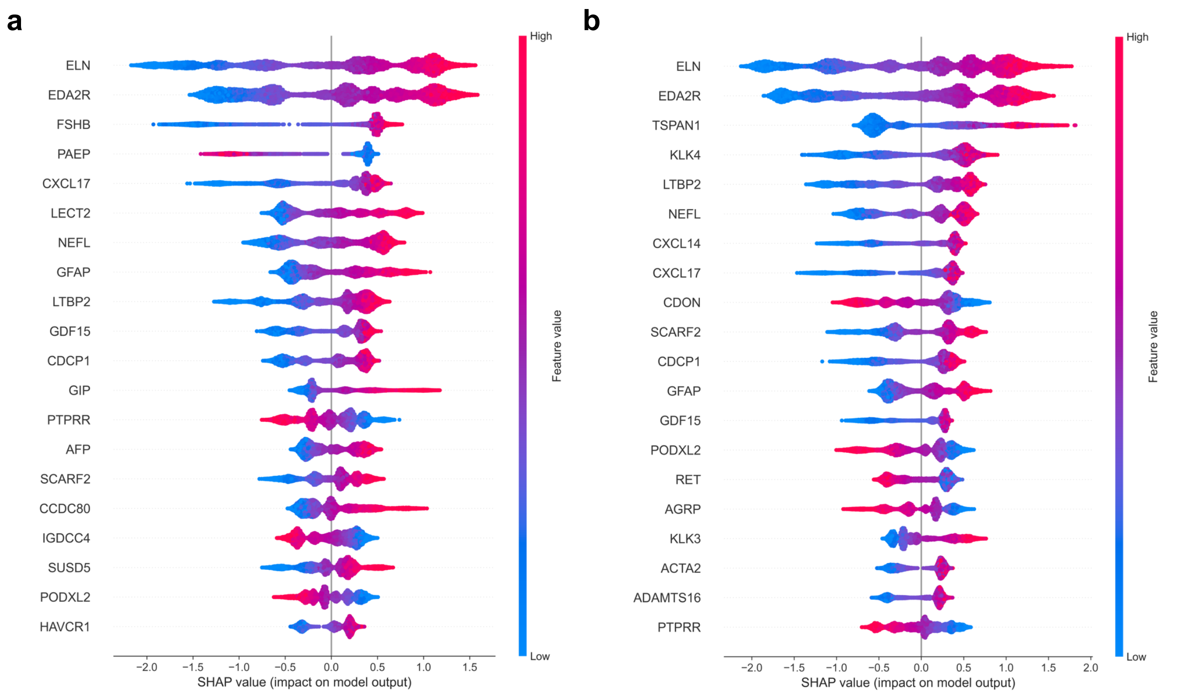

Fig. S9. Top 20 most important proteins for predicting age in females and males. Comparison of the top 20 most important proteins for predicting chronological age for **a)** females (n=7,374) and **b)** males (n=6,259). Protein importance was calculated for each protein by taking the mean of the absolute SHAP values across all participants for that protein. The x-axis shows the SHAP values from the sex-specific LightGBM model for each protein, with the SHAP value for each individual participant as an individual dot. The sign of SHAP values represents the effect on the model outcome (i.e., SHAP values > 0 indicate predicting an older age and SHAP values < 0 indicate predicting a younger age). Dots are color coded according to the value of the protein feature (red indicates higher protein expression and blue indicates lower protein expression). An example of how to read: higher protein expression of the ELN protein predicts higher age and lower protein expression of PODXL2 predicts higher age.

Supplementary Table Descriptions

Table S1. All age-associated proteins (APs) in the full 204-protein proteomic age clock. A list of all 204 proteins identified in our proteomic aging model. Further included are the UniProt ID for each protein, as well as summary information on whether each protein was identified in previous proteomic clocks or whether the corresponding gene was identified in previous DNA methylation clocks. An entry of "1" in columns D-I indicates overlap with these clocks, and "0" indicates no overlap. Proteomic clock comparisons include Johnson et al. 2020,[1] Coenen et al. 2023,[2] and Lehallier et al. 2019.[3] Methylation clock comparisons include the Horvath clock,[4] PhenoAge,[5] and DunedinPACE.[6]

Table S2. 20 age-associated proteins (APs) in the ProtAge20 clock. A list of all 20 proteins identified in our 20-protein proteomic aging model. Further included are the UniProt ID for each protein, as well as summary information on whether each protein was identified in previous proteomic clocks or whether the corresponding gene was identified in previous DNA methylation clocks.

Table S3. Associations between ProtAgeGap (PAG) and biological aging / functional status outcomes (all UK Biobank participants, n=45,441). Summary statistics from linear/logistic regressions between ProtAgeGap and all aging biomarkers and measures of physical and cognitive function tested. P-values are corrected for multiple comparisons using the false discovery rate (FDR).

Table S4. Associations between ProtAgeGap (PAG) and biological aging / functional status outcomes (healthy UK Biobank participants, n=20,353). Summary statistics from linear/logistic regressions between ProtAgeGap and all aging biomarkers and measures of physical and cognitive function tested. P-values are corrected for multiple comparisons using the false discovery rate (FDR).

Table S5. Associations between ProtAgeGap20 (PAG20) and biological aging / functional status outcomes (all UK Biobank participants, n=45,441) Summary statistics from linear/logistic regressions between ProtAgeGap and all aging biomarkers and measures of physical and cognitive function tested. P-values are corrected for multiple comparisons using the false discovery rate (FDR).

Table S6. Associations between ProtAgeGap20 (PAG20) and biological aging / functional status outcomes (healthy UK Biobank participants, n=20,353). Summary statistics from linear/logistic regressions between ProtAgeGap and all aging biomarkers and measures of physical and cognitive function tested. P-values are corrected for multiple comparisons using the false discovery rate (FDR).

Table S7. Associations between ProtAgeGap and mortality and incident non-cancer diseases (Model 1) in the full UK Biobank population (n=45,441). Summary statistics from Cox proportional hazards models between ProtAgeGap and all-cause mortality and incidence of all non-cancer illnesses using model 1 covariates (age and sex). P-values are corrected for multiple comparisons using the false discovery rate (FDR).

Table S8. Associations between ProtAgeGap and mortality and incident non-cancer diseases (Model 2) in the full UK Biobank population (n=45,441). Summary statistics from Cox proportional hazards models between ProtAgeGap and all-cause mortality and incidence of all non-cancer illnesses using model 2 covariates (age, sex, ethnicity, Townsend deprivation index, recruitment centre, IPAQ activity group, and smoking status). P-values are corrected for multiple comparisons using the false discovery rate (FDR).

Table S9. Associations between ProtAgeGap and mortality and incident non-cancer diseases (Model 3) in the full UK Biobank population (n=45,441). Summary statistics from Cox proportional hazards models between ProtAgeGap and all-cause mortality and incidence of all non-cancer illnesses using model 2 covariates (age, sex, ethnicity, Townsend deprivation index, recruitment centre, IPAQ activity group, smoking status, BMI, and prevalent hypertension). P-values are corrected for multiple comparisons using the false discovery rate (FDR).

Table S10. Associations between ProtAgeGap20 and mortality and incident non-cancer diseases (Model 1) in the full UK Biobank population (n=45,441). Summary statistics from Cox proportional hazards models between ProtAgeGap and all-cause mortality and incidence of all non-cancer illnesses using model 1 covariates (age and sex). P-values are corrected for multiple comparisons using the false discovery rate (FDR).

Table S11. Associations between ProtAgeGap20 and mortality and incident non-cancer diseases (Model 2) in the full UK Biobank population (n=45,441). Summary statistics from Cox proportional hazards models between ProtAgeGap20 and all-cause mortality and incidence of all non-cancer illnesses using model 2 covariates (age, sex, ethnicity, Townsend deprivation index, recruitment centre, IPAQ activity group, and smoking status). P-values are corrected for multiple comparisons using the false discovery rate (FDR).

Table S12. Associations between ProtAgeGap20 and mortality and incident non-cancer diseases (Model 3) in the full UK Biobank population (n=45,441). Summary statistics from Cox proportional hazards models between ProtAgeGap20 and all-cause mortality and incidence of all non-cancer illnesses using model 2 covariates (age, sex, ethnicity, Townsend deprivation index, recruitment centre, IPAQ activity group, smoking status, BMI, and prevalent hypertension). P-values are corrected for multiple comparisons using the false discovery rate (FDR).

Table S13. Associations between ProtAgeGap and incident cancers (Model 1) in the full UK Biobank population (n=45,441). Summary statistics from Cox proportional hazards models between ProtAgeGap and all-cause mortality and incidence of cancers using model 1 covariates (age and sex). P-values are corrected for multiple comparisons using the false discovery rate (FDR).

Table S14. Associations between ProtAgeGap and incident cancers (Model 2) in the full UK Biobank population (n=45,441). Summary statistics from Cox proportional hazards models between ProtAgeGap and all-cause mortality and incidence of cancers using model 2 covariates (age, sex, ethnicity, Townsend deprivation index, recruitment centre, IPAQ activity group, and smoking status). P-values are corrected for multiple comparisons using the false discovery rate (FDR).

Table S15. Associations between ProtAgeGap and incident cancers (Model 3) in the full UK Biobank population (n=45,441). Summary statistics from Cox proportional hazards models between ProtAgeGap and all-cause mortality and incidence of cancers using model 2 covariates (age, sex, ethnicity, Townsend deprivation index, recruitment centre, IPAQ activity group, smoking status, BMI, and prevalent hypertension). P-values are corrected for multiple comparisons using the false discovery rate (FDR).

Table S16. Age-specific incidence rates in the UK Biobank for mortality and age-related diseases by ProtAgeGap (PAG) deciles. Cumulative incidence rates are shown for those who are aged 50, 55, 60, and 65 years at recruitment in the UK Biobank (n=45,441). Incidence rates are for the 11-16 years after recruitment in the UK Biobank.

Table S17. Age-specific incidence rates in the China Kadoorie Biobank for mortality and age-related diseases by ProtAgeGap (PAG) deciles. Cumulative incidence rates are shown for those who are aged 35, 40, 45, 50, 55, 60, and 65 years at recruitment in the China Kadoorie Biobank (n=2,026). Incidence rates are for the 11-14 years after recruitment in the China Kadoorie Biobank.

Table S18. Individual aging biomarker and frailty variables tested in the UK Biobank. Descriptions and Field IDs for variables used in aging biomarker and functional outcome analyses.

Table S19. Items used to construct the frailty index in the UK Biobank. Descriptions and Field IDs for variables used to construct the summary frailty index.

Table S20. Variables used to calculate prevalence and incidence of chronic diseases and clinical risk factors in the UK Biobank. ICD-9/10 codes and descriptions of self-report, biochemistry, and clinical interview variables used to code prevalent and incident disease outcomes.

Table S21. Variables used to calculate prevalence and incidence of chronic diseases and clinical risk factors in the China Kadoorie Biobank. ICD-10 codes used to code incident disease outcomes.

References

1 Johnson, A. A., Shokhirev, M. N., Wyss-Coray, T. & Lehallier, B. Systematic review and analysis of human proteomics aging studies unveils a novel proteomic aging clock and identifies key processes that change with age. *Ageing Res Rev* **60**, 101070 (2020). https://doi.org/10.1016/j.arr.2020.101070

2 Coenen, L., Lehallier, B., de Vries, H. E. & Middeldorp, J. Markers of aging: Unsupervised integrated analyses of the human plasma proteome. *Front Aging* **4**, 1112109 (2023). https://doi.org/10.3389/fragi.2023.1112109

3 Lehallier, B. *et al.* Undulating changes in human plasma proteome profiles across the lifespan. *Nat Med* **25**, 1843-1850 (2019). https://doi.org/10.1038/s41591-019-0673-2

4 Horvath, S. DNA methylation age of human tissues and cell types. *Genome Biol* **14**, R115 (2013). https://doi.org/10.1186/gb-2013-14-10-r115

5 Levine, M. E. *et al.* An epigenetic biomarker of aging for lifespan and healthspan. *Aging (Albany NY)* **10**, 573-591 (2018). https://doi.org/10.18632/aging.101414

6 Belsky, D. W. *et al.* DunedinPACE, a DNA methylation biomarker of the pace of aging. *Elife* **11** (2022). https://doi.org/10.7554/eLife.73420
