# Supplementary material for: Proteomic aging clock predicts mortality and risk of common age-related diseases in diverse populations
Source: Nat Med. 2024 Aug 8;30(9):2450–60. doi: 10.1038/s41591-024-03164-7 (PMC11405266; doi:10.1038/s41591-024-03164-7)
Supplement: Supplementary file 2 — Reporting Summary [file 41591_2024_3164_MOESM2_ESM.pdf]

nature portfolio

Corresponding author(s): M. Austin Argentieri, Cornelia M. van Duijn

Last updated by author(s): Jun 17, 2024

Reporting Summary

Nature Portfolio wishes to improve the reproducibility of the work that we publish. This form provides structure for consistency and transparency in reporting. For further information on Nature Portfolio policies, see our Editorial Policies and the Editorial Policy Checklist.

Statistics

For all statistical analyses, confirm that the following items are present in the figure legend, table legend, main text, or Methods section.

n/a	Confirmed	
☐	☒	The exact sample size (*n*) for each experimental group/condition, given as a discrete number and unit of measurement
☐	☒	A statement on whether measurements were taken from distinct samples or whether the same sample was measured repeatedly
☐	☒	The statistical test(s) used AND whether they are one- or two-sided<br>*Only common tests should be described solely by name; describe more complex techniques in the Methods section.*
☐	☒	A description of all covariates tested
☐	☒	A description of any assumptions or corrections, such as tests of normality and adjustment for multiple comparisons
☐	☒	A full description of the statistical parameters including central tendency (e.g. means) or other basic estimates (e.g. regression coefficient) AND variation (e.g. standard deviation) or associated estimates of uncertainty (e.g. confidence intervals)
☐	☒	For null hypothesis testing, the test statistic (e.g. *F*, *t*, *r*) with confidence intervals, effect sizes, degrees of freedom and *P* value noted<br>*Give P values as exact values whenever suitable.*
☒	☐	For Bayesian analysis, information on the choice of priors and Markov chain Monte Carlo settings
☒	☐	For hierarchical and complex designs, identification of the appropriate level for tests and full reporting of outcomes
☐	☒	Estimates of effect sizes (e.g. Cohen's *d*, Pearson's *r*), indicating how they were calculated

Our web collection on statistics for biologists contains articles on many of the points above.

Software and code

Policy information about availability of computer code

Data collection	No software was used - data were already collected.
Data analysis	All statistical analyses were carried out using Python v.3.6 and R v.4.2.2. Analysis code and two requirements.txt files for R and Python with all statistical package versions used are available at https://github.com/miargentieri/proteomic-age-ukb.

For manuscripts utilizing custom algorithms or software that are central to the research but not yet described in published literature, software must be made available to editors and reviewers. We strongly encourage code deposition in a community repository (e.g. GitHub). See the Nature Portfolio guidelines for submitting code & software for further information.

Data

Policy information about availability of data

All manuscripts must include a data availability statement. This statement should provide the following information, where applicable:

- Accession codes, unique identifiers, or web links for publicly available datasets
- A description of any restrictions on data availability
- For clinical datasets or third party data, please ensure that the statement adheres to our policy

UK Biobank data are available through a procedure described at: https://www.ukbiobank.ac.uk/enable-your-research.

The China Kadoorie Biobank (CKB) is a global resource for the investigation of lifestyle, environmental, blood biochemical and genetic factors as determinants of

common diseases. The CKB study group is committed to making the cohort data available to the scientific community in China, the UK, and worldwide to advance knowledge about the causes, prevention and treatment of disease. For detailed information on what data is currently available to open access users and how to apply for it, please visit: https://www.ckbiobank.org/data-access. A research proposal will be requested to ensure that any analysis is performed by bona fide researchers. Researchers who are interested in obtaining additional information or data that underlines this paper should contact: ckbaccess@ndph.ox.ac.uk. For any data that are not currently available via open access, researchers may need to develop formal collaboration with the CKB study group.

FinnGen data can be accessed through the Fingenious® services (https://site.fingenious.fi/en/) managed by FINBB. Finnish Health register data can be applied from Findata (https://findata.fi/en/data/).

Experimental protein-protein interaction information used from the STRING database (v.12) can be accessed programmatically using the STRING API (https://string-db.org/api) or can be downloaded directly from the STRING website (https://string-db.org/cgi/download.pl).

Research involving human participants, their data, or biological material

Policy information about studies with human participants or human data. See also policy information about sex, gender (identity/presentation), and sexual orientation and race, ethnicity and racism.

Reporting on sex and gender

Sex-specific proteomic age clocks were first calculated to test for sex-specific performance differences. None were found, and so the final proteomic age clock was calculated in both sexes combined. Distributions of proteomic aging acceleration according to sex is reported in the UK Biobank (UKB), China Kadoorie Biobank (CKB), and FinnGen. Distributions did not show major differences between the two groups. In all regression analyses, sex was used as a covariate. Cumulative incidence of mortality and incident diseases according to accelerated proteomic aging was tested in all participants as well as separately in both sexes. Sex-specific models are reported in Fig. S6-7.

Reporting on race, ethnicity, or other socially relevant groupings

Self-reported ethnicity was used as a covariate in regression models. Further, distributions of proteomic aging acceleration according to self-reported ethnicity (UKB) and geographic region of recruitment (CKB) are reported in Fig. 2.

Population characteristics

We used plasma proteomic expression data from the subset of 45,441 randomly selected UKB participants (54% female, age range: 39-71 years), 3,977 Chinese (CKB) participants in an ischemic heart disease (IHD) case-cohort study (54% female, age range: 30-78 years), and 1,990 Finnish (FinnGen) participants (52% female, age range: 19-78 years). Across 11-16 years of follow-up in the UKB and 11-14 years of follow-up in the CKB, there were 4,828 (10.6%) and 1,426 (36%) deaths, respectively. Proteomic profiling was conducted among mostly healthy participants in FinnGen without major diseases and only 1% (n=22) died during follow up.

Recruitment

Participants were recruited to the UKB between 2006-2010. Further information on UKB recruitment and data collection has been published previously (https://www.ukbiobank.ac.uk/media/gnkeyh2q/study-rationale.pdf). CKB participants were recruited from ten geographically diverse (five rural and five urban) areas across China during 2004-2008. Information on CKB recruitment and data collection has been previously reported (https://doi.org/10.1093/ije/dyr120). FinnGen includes 9 Finnish biobanks, research institutes, universities and university hospitals, 13 international pharmaceutical industry partners and the Finnish Biobank Cooperative (FINBB). The project utilizes data from the nationwide longitudinal health register collected since 1969 from every resident in Finland.

Ethics oversight

UK Biobank data use (Project Application Number 61054) was approved by the UK Biobank according to their established access procedures. UK Biobank has approval from the North West Multi-centre Research Ethics Committee (MREC) as a Research Tissue Bank (RTB), and as such researchers using UK Biobank data do not require separate ethical clearance and can operate under the RTB approval. The China Kadoorie Biobank (CKB) complies with all the required ethical standards for medical research on human subjects. Ethical approvals were granted and have been maintained by the relevant institutional ethical research committees in the UK and China. Study subjects in FinnGen provided informed consent for biobank research, based on the Finnish Biobank Act. The FinnGen study is approved by Finnish Institute for Health and Welfare (permit numbers: THL/2031/6.02.00/2017, THL/1101/5.05.00/2017, THL/341/6.02.00/2018, THL/2222/6.02.00/2018, THL/283/6.02.00/2019, THL/1721/5.05.00/2019 and THL/1524/5.05.00/2020), Digital and population data service agency (permit numbers: VRK43431/2017-3, VRK/6909/2018-3, VRK/4415/2019-3), the Social Insurance Institution (permit numbers: KELA 58/522/2017, KELA 131/522/2018, KELA 70/522/2019, KELA 98/522/2019, KELA 134/522/2019, KELA 138/522/2019, KELA 2/522/2020, KELA 16/522/2020), Findata permit numbers THL/2364/14.02/2020, THL/4055/14.06.00/2020, THL/3433/14.06.00/2020, THL/4432/14.06/2020, THL/5189/14.06/2020, THL/5894/14.06.00/2020, THL/6619/14.06.00/2020, THL/209/14.06.00/2021, THL/688/14.06.00/2021, THL/1284/14.06.00/2021, THL/1965/14.06.00/2021, THL/5546/14.02.00/2020, THL/2658/14.06.00/2021, THL/4235/14.06.00/2021, Statistics Finland (permit numbers: TK-53-1041-17 and TK/143/07.03.00/2020 (earlier TK-53-90-20) TK/1735/07.03.00/2021, TK/3112/07.03.00/2021) and Finnish Registry for Kidney Diseases permission/extract from the meeting minutes on 4th July 2019.

Note that full information on the approval of the study protocol must also be provided in the manuscript.

Field-specific reporting

Please select the one below that is the best fit for your research. If you are not sure, read the appropriate sections before making your selection.

☐ Life sciences ☒ Behavioural & social sciences ☐ Ecological, evolutionary & environmental sciences

For a reference copy of the document with all sections, see nature.com/documents/nr-reporting-summary-flat.pdf

Behavioural & social sciences study design

All studies must disclose on these points even when the disclosure is negative.

Study description	The study used quantitative methods within the context of prospective cohort study data. We employed a machine learning model (LightGBM) that uses plasma proteomic data generated using the Olink Explore 3072 platform in the UK Biobank (n=45,441) to predict chronological age. Independent data from the China Kadoorie Biobank (CKB) and FinnGen biobank were used for further independent validation of the proteomic age clock model. Protein predicted age (ProtAge) was calculated in the full UKB sample using 5-fold cross-validation and LightGBM. Proteomic age gap (ProtAgeGap) was calculated as the difference between ProtAge and chronological age. We used linear and logistic regression to test associations between ProtAgeGap and a comprehensive panel of biological aging markers and measures of frailty and physical/cognitive status. Further, we used Cox proportional hazards models to test associations between ProtAgeGap and mortality, 14 common diseases, and 12 cancers.
Research sample	Our study uses secondary data from 45,441 UK Biobank (UKB, 54% female, age range: 39-71 years), 3,977 Chinese (CKB) participants in an ischemic heart disease (IHD) case-cohort study (54% female, age range: 30-78 years), and 1,990 Finnish (FinnGen) participants (52% female, age range: 19-78 years). We chose the UKB, CKB, and FinnGen or the breadth of exposures, phenotypes, incident disease diagnosis data, and biological data available in each, allowing for comprehensive and integrative modeling. We used previously collected self-report questionnaire data, data from clinical interviews, biochemical measures from blood samples collected at baseline, and hospital diagnosis and mortality information from linked inpatient and mortality register data. UKB proteomics samples were randomly selected to be roughly representative of the full UKB cohort. CKB proteomics samples are not representative of the full CKB cohort due to the nested case-cohort design. FinnGen proteomics samples are not representative of the full FinnGen cohort since they were chosen as healthy participants with little morbidity or mortality during follow up.
Sampling strategy	The final sample included 45,441 UKB participants, 3,977 CKB participants, and 1,990 FinnGen participants. We randomly split the UKB cohort into 70% training and 30% test sets to develop the proteomic age clock. In the training phase, we trained the proteomic age clock model to predict chronological age using normalized expression of 2,897 proteins from the Olink Explore 3027 panel. Models were then validated in the CKB and FinnGen participants. Associations between proteomic age gap and health outcomes and aging phenotypes were tested in the full UKB (n=45,441) and CKB (n=3,977) samples. Subsampling within each cohort to select participants for proteomic analysis was performed previously by the cohorts and not by our study team. UKB proteomics samples were randomly selected to be roughly representative of the full UKB cohort. CKB proteomics samples were selected using a nested case-cohort design to be powered for ischemic heart disease analyses. FinnGen proteomics samples are not representative of the full FinnGen cohort since they were chosen as healthy participants with little morbidity or mortality during follow up. Power analyses were not conducted for this study - we used all participants with proteomics data avaialable in each cohort. With the exception of UKB, where we excluded roughly 7,000 participants who were not randomly selected and instead selected by pharma partners to be enriched for different disease phenotypes.
Data collection	Participants were recruited to the UKB between 2006-2010. Further information on UKB recruitment and data collection has been published previously (https://www.ukbiobank.ac.uk/media/gnkeyh2q/study-rationale.pdf). CKB participants were recruited from ten geographically diverse (five rural and five urban) areas across China during 2004-2008. Information on CKB recruitment and data collection has been previously reported (https://doi.org/10.1093/ije/dyr120). FinnGen includes 9 Finnish biobanks, research institutes, universities and university hospitals, 13 international pharmaceutical industry partners and the Finnish Biobank Cooperative (FINBB). The project utilizes data from the nationwide longitudinal health register collected since 1969 from every resident in Finland. No new data were collected from UKB, CKB, or FinnGen participants for this study. Researchers in our study were not blinded to the study hypothesis.
Timing	Baseline UKB collection took place from March 15 2006 until September 27 2010. Follow up mortality and incident disease data were collected until December 2 2022, leaving 11-15 years of follow up in the UKB. Baseline CKB collection took place from 2004-2008 (precide dates not reported in dataset). Follow up mortality and incident disease data were collected until January 2019, leaving 11-14 years of follow up. No follow up data were used for FinnGen.
Data exclusions	We restricted our UKB sample to those participants with Olink Explore data available at baseline who were randomly sampled from the main UKB population (n=45,441). We restricted our CKB and FinnGen sample to those participants with Olink Explore data available.
Non-participation	No participants dropped out during the course of our study.
Randomization	Participants were randomly assigned to training and test sets for completing machine learning analyses.

Reporting for specific materials, systems and methods

We require information from authors about some types of materials, experimental systems and methods used in many studies. Here, indicate whether each material, system or method listed is relevant to your study. If you are not sure if a list item applies to your research, read the appropriate section before selecting a response.

Materials & experimental systems

n/a	Involved in the study
☒	☐ Antibodies
☒	☐ Eukaryotic cell lines
☒	☐ Palaeontology and archaeology
☒	☐ Animals and other organisms
☒	☐ Clinical data
☒	☐ Dual use research of concern
☒	☐ Plants

Methods

n/a	Involved in the study
☒	☐ ChIP-seq
☒	☐ Flow cytometry
☒	☐ MRI-based neuroimaging
